# Catalytically Active Light Printed Microstructures

**DOI:** 10.1002/adma.202506663

**Published:** 2025-06-06

**Authors:** Alicia K. Finch, Sebastian Gillhuber, Hendrik Frisch, Peter W. Roesky, Christopher Barner‐Kowollik

**Affiliations:** ^1^ School of Chemistry and Physics Centre for Materials Science Queensland University of Technology (QUT) 2 George Street Brisbane QLD 4000 Australia; ^2^ Institute of Nanotechnology (INT) Karlsruhe Institute of Technology (KIT) Hermann‐von‐Helmholtz‐Platz 1 76344 Eggenstein‐Leopoldshafen Germany; ^3^ Institute of Functional Interfaces (IFG) Karlsruhe Institute of Technology (KIT) Hermann‐von‐Helmholtz‐Platz 1 76344 Eggenstein‐Leopoldshafen Germany; ^4^ Institute of Inorganic Chemistry Karlsruhe Institute of Technology (KIT) Engesserstraße 15 76131 Karlsruhe Germany

**Keywords:** 3D printing, catalytically active materials, direct laser writing, functional materials, multi‐materials, photocatalysis, stereolithography

## Abstract

Light‐induced additive manufacturing (3D printing) has revolutionized manufacturing and its integration into the fabrication of catalysts holds key potential to enable facile access to optimized catalyst geometries and designs. Herein – for the first time – micro‐ and macro‐sized photocatalytically active 3D printed objects are introduced via a dual‐function photoresin using a ruthenium(II) complex containing monomer as both a photoinitiator for the 3D printing process and as the active photocatalyst within the printed structure. The approach leverages the spatial and temporal control afforded by light‐induced 3D printing techniques during both one‐ and two‐photon printing to precisely position the photocatalyst within intricate geometries using a pentaerythritol triacrylate (PETA) based resin. The successful incorporation of ruthenium(II) complexes is demonstrated via time‐of‐flight secondary‐ion mass spectrometry (ToF‐SIMS) into desired sections of 3D‐printed objects. The one‐ and two‐photon fabricated architectures show photocatalytic activity in the C─H arylation of activated aryl bromides. The potential of tailored catalytically active 3D objects is exemplified by one of the microscale designs. This design, utilizing only 1% of the volume of a macroscale structure fabricated from the same resin, achieved 75% of the photocatalytic performance.

## Graphical abstract

The fabrication of micro‐ and macro‐scale 3D‐printed multi‐material structures incorporating photocatalytically active Ru(II) sites using a dual‐function photoresin is introduced. The system utilizes a Ru(II)‐functionalized monomer, enabling the assembly of 3D structures with commercially available crosslinkers. The catalytic activity is demonstrated through the C─H arylation of activated aryl bromides.

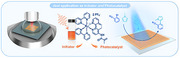

## 1 Introduction

In recent decades the chemical industry has undergone a significant transformation in catalyst utilization. Traditional large‐scale chemical plants are increasingly replaced by advanced, more compact reactors.^[^ ^1^ ^]^ The shift is driven by a strong focus on energy efficiency, lowering the usage of precious metals, and optimized resource management to minimize waste and unwanted byproducts.^[^ ^1^, ^2^ ^]^ The design of highly efficient catalysts on the microscale necessitates a departure from traditional fabrication techniques such as impregnation,^[^ ^3^ ^]^ precipitation,^[^ ^4^ ^]^ adsorption,^[^ ^5^ ^]^ sol‐gel,^[^ ^6^ ^]^ spray drying^[^ ^7^ ^]^ and hydrothermal synthesis,^[^ ^8^ ^]^ which often face limitations in design freedom and precision.^[^ ^1^, ^9^ ^]^ 3D microfabrication has emerged as a front‐runner in the fabrication of catalysts as it facilitates the fabrication of complex geometries that are otherwise inaccessible.^[^ ^10^ ^]^ Among the various 3D printing technologies, photochemical approaches stand out for their ability to produce highly defined structures.^[^ ^11^ ^]^ Charles Hull pioneered 3D printing by developing stereolithography in 1986, marking the first experimental realization of light‐induced 3D printing techniques.^[^ ^12^ ^]^ Here, free‐form additive manufacturing is realized through the precise control of a laser beam, which solidifies the resin in a controlled, layer‐by‐layer fashion. The linear relationship between optical intensity and the photocuring reaction results in rapid solidification within seconds, enabling the efficient layer‐by‐layer construction of the 3D object.^[^ ^13^ ^]^ Higher printing resolution can be achieved through two‐photon printing – often termed Direct Laser Writing (DLW) – a versatile technique for fabricating near‐arbitrary shaped entities on the nano‐ and microscale.^[^ ^14^ ^]^ During DLW, a pulsed femtosecond laser beam is precisely focused into a photosensitive liquid material, initiating a photochemical crosslinking process through two‐photon absorption (2PA).^[^ ^11^, ^15^ ^]^ The precise control of the laser's focal point ensures that only a small volume element receives sufficient irradiation for 2PA to occur, enabling DLW to achieve sub‐micrometer resolution.^[^ ^16^ ^]^ Furthermore, long‐wavelength excitation sources allow for deeper penetration into the resin, reaching depths of several tens of micrometers.^[^ ^17^ ^]^ A photocurable resin typically consists of a multi‐functional monomer (e.g., trifunctional crosslinker, pentaerythritol triacrylate, PETA) and a photoinitiator. However, using such classical photoresists results in objects with no or limited functionality.^[^ ^18^ ^]^ Integrating catalytic activity with light‐induced 3D printing techniques to fabricate functional architectures presents significant challenges.

While 3D printing has been explored for organometallic and organic catalysts, its application to photocatalysts is limited.^[^ ^19^ ^]^ Current fabrication methods for 3D‐printed photocatalysts primarily rely on post‐processing techniques, such as depositing catalysts onto the printed structure or incorporating covalent binding sites.^[^ ^20^ ^]^ Such multi‐step approaches increase fabrication time and potentially introduce defects that could compromise mechanical properties.^[^ ^1^ ^]^ To print photocatalytically active objects directly, catalysts have been dispersed within the printing resin, however, this strategy could potentially cause leaching of the catalyst and lead to activity loss.^[^ ^21^ ^]^ To prevent leaching, photocatalysts with monomeric functionalization can be covalently embedded into the formed polymer network upon polymerization. Such a strategy has been implemented by Cormier and coworkers using an Eosin Y‐based monomer digital light processing to integrate 3 wt.% of Eosin Y covalently in the printed object.^[^ ^22^ ^]^ While this elegant example demonstrates successful 3D printing of an organic photocatalyst, it is based on resin formulations containing two photoreactive species: the desired photocatalyst and a photoinitiator to enable the curing of the resin. Besides the more complex resin formulation, unreacted photoinitiator may be entrapped within the final 3D‐printed structure. The resulting presence of two light‐reactive species may induce undesired side reactions upon irradiation, potentially compromising the selectivity of the catalytic process.

Herein, we not only introduce micro‐sized catalytically active 3D objects but address this key challenge by utilizing a single monomeric photoreactive species, concomitantly serving as a photoinitiator for the 3D printing process and imparting photocatalytic activity to the printed structure. We propose the use of stable ruthenium polypyridyl complexes, given their wide applications in catalysis and reported efficacy as photoinitiators for conventional light‐induced printing approaches such as stereolithography (SLA) as demonstrated by systems employing tris(2,2′‐bipyridine) dichloro‐ruthenium(II) hexahydrate and sodium persulfate.^[^ ^23^ ^]^

While the application of ruthenium polypyridyl complexes in two‐photon printing has been less explored,^[^ ^17a^ ^]^ Malval and coworkers have successfully applied [RuL(2,2′‐bipyridine)_2_]^2+^ (L  =  functionalized bipyridines) heteroleptic complexes for direct laser writing of electrochemiluminescent 2D structures.^[^ ^24^ ^]^

Herein, the monomeric Ru(II)‐complex design is based on a phenanthroline ligand functionalized with acrylamide to enable the covalent incorporation of a Ru(II)‐complex into the polymer network without drastically affecting the ligand upon polymerization. The additive manufacturing technologies DLW and SLA are used to show that the manufacturing is possible via one‐ and two‐photon printing, with the initiation of the curing process occurring through the Ru(II)‐complex, resulting in the first micro‐ and macro‐sized photocatalytically active 3D printed objects from a dual function photoresin. Finally, we explore the catalytic performance of structures obtained from both printing techniques, using the C−H arylation of an activated aryl bromide as a representative example.

## 2 Results and Discussion

**Scheme 1:**
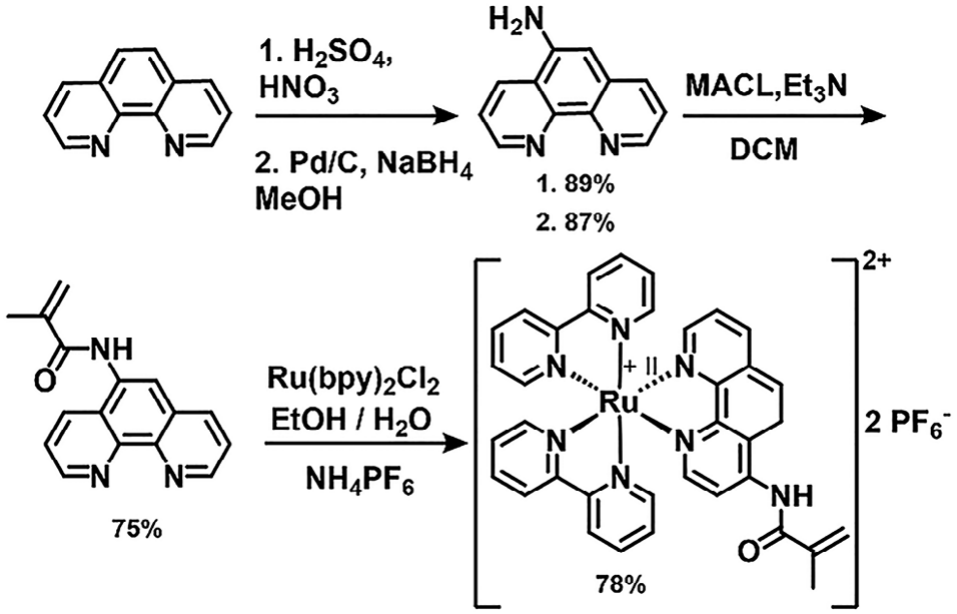
Synthesis of the Ru(II)‐complex containing monomer (**Ru(II)‐CM**), bpy = 2,2′‐bipyridine, MACL = methacrylic chloride.

The photoresist design is based on a commercially available trifunctional crosslinker, PETA, offering several benefits, including a rapid crosslinking reaction, convenient handling due to its liquid state and heat resistance, and the ability to generate stable, robust materials suitable for intricate structures.^[^ ^18^, ^25^ ^]^ These properties simplify photoresist formulation as other components, such as the functionality‐providing complex in solid form, can readily be dissolved in the resin by applying mild heat. The synthesis of the Ru(II)‐complex monomer (**Ru(II)‐CM**, **Scheme** **1**) necessitates the prior preparation of a ligand incorporating a polymerizable moiety. For the synthesis of the acrylamide‐based monomer, 1,10‐phenanthroline was regioselectively nitrated and reduced to 5‐amino‐1,10‐phenanthroline following a modified literature procedure.^[^ ^26^ ^]^ Subsequently, 5‐amino‐1,10‐phenanthroline was coupled with methacryloyl chloride to afford *N*‐(1,10‐phenanthrolin‐5‐yl) methacrylamide, requiring no further purification. The Ru(II)‐complex was prepared using a modified literature procedure of Zabarska et al., readily forming bis(bpy)(1,10‐phenanthrolin‐5‐yl)acrylamide) ruthenium(II) hexafluorophosphate (**Ru(II)‐CM**) via a ligand exchange (bpy, 2,2′‐bipyridine).^[^ ^27^ ^]^ The resulting **Ru(II)‐CM** serves as the functional component in the PETA‐based resin. Notably, following the above approach, the catalytically active complex is covalently anchored within the printed structure, enhancing stability and minimizing catalyst leaching.^[^ ^28^ ^]^ This was further confirmed by printing with both **Ru(II)‐CM** and tris(bipyridine)ruthenium(II) hexafluorophosphate in identical resin formulations (refer to Section S2.5 of Supporting Information). The tris(bipyridine)ruthenium(II) hexafluorophosphate did not dissolve as well as the **Ru(II)‐CM** in the PETA and propylene carbonate mixture, resulting in dispersed crystals, visible during the printing process, as illustrated in Figure S5 (Supporting Information).

**Figure 1:**
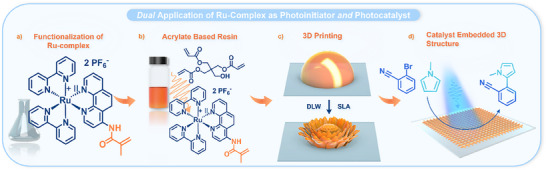
Overview of the present work. a) A Ru(II)‐complex equipped with a methacrylamide moiety. b) Resin formulation based on PETA with the Ru(II)‐complex as a co‐monomer and initiator. c) 3D printing of Ru(II)‐containing structures via direct laser writing or stereolithography. d) Use of the printed structures as solid‐state catalysts. During the writing process, the Ru(II)‐complex enables the crosslinking reaction. Critically, the Ru(II)‐complex integrated into the structure acts as a photocatalyst in the C─H arylation of activated aryl bromides.

We initially explored 3D printing via DLW and later investigated SLA, both employing identical resin formulations (**Figure** **1**b). Specifically, we systematically evaluated various formulations of the resin by adjusting the ratio of the **Ru(II)‐CM** to the trifunctional crosslinker PETA. The resist also contained 4 wt.% propylene carbonate to increase the solubility of the **Ru(II)‐CM**. We found a 20 wt.% **Ru(II)‐CM** loading to be the maximum achievable, yielding highly defined and stable structures. At higher **Ru(II)‐CM** concentrations, insufficient light penetration hindered printing, and no structures were obtained with higher loadings (refer to Section S2.5. in the Supporting Information). Thus, all microstructures presented herein, unless otherwise specified, were fabricated using a resist formulation of identical composition (resin composed of 20 wt.% **Ru(II)‐CM**, 80 wt.% PETA, formulated with an additional 4 wt.% propylene carbonate) and printed onto glass coverslips. Covalent attachment of the scaffold to the coverslip was facilitated by prior silanization of the coverslip with 3‐(trimethoxysilyl)propyl methacrylate. Following the printing process, the glass slides were immersed in acetone to remove any residual non‐polymerized material.

**Figure 2:**
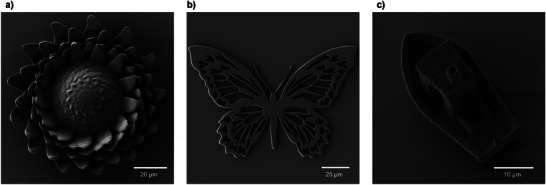
a, b) SEM images of printed structures with high complexity, printed with the resin of 20 wt.% **Ru(II)‐CM** to 80 wt.% PETA via DLW. A writing speed of 10 000 µm s^−1^ and a laser power of 35 mW was utilized. c) SEM image of a #3DBenchy structure written at 6000 µm s^−1^ and a laser power of 35 mW also printed with the resist containing a ratio of 20 wt.% **Ru(II)‐CM** to 80 wt.% PETA.

To optimize the writing conditions, we varied key parameters during the writing process and monitored their impact on the definition of the resulting 3D structures. To facilitate the analysis, we fabricated arrays of blocks (Figure S2, Supporting Information), each written using a distinct set of parameters. The selected laser powers ranged between 20 and 40 mW, while the writing speed varied between 2500 and 20 000 µm s^−1^. Outside this range, either no writing was observed, the excessive laser exposure led to micro‐explosions or to defective structures depending on the complexity. Through the aforementioned printing tests, the photoinitiation efficiency of other commercial resists was compared, using a dimensionless Figure‐of‐Merit (FOM) applicable across all experiments on a given photoresist.^[^ ^29^ ^]^ A FOM of 53 was calculated and is comparable to the FOM of a mixture of commercial Irgacure 369 and PETA of 56.1 (refer to Section S2.5, Supporting Information). Based on the evaluation of the printed blocks and their fabrication quality via scanning electron microscopy (SEM), more complex structures were printed using a laser power of 35 mW, as shown in **Figure** **2**a–c with various writing speeds ranging from 6000 to 10 000 µm s^−1^.

**Figure 3:**
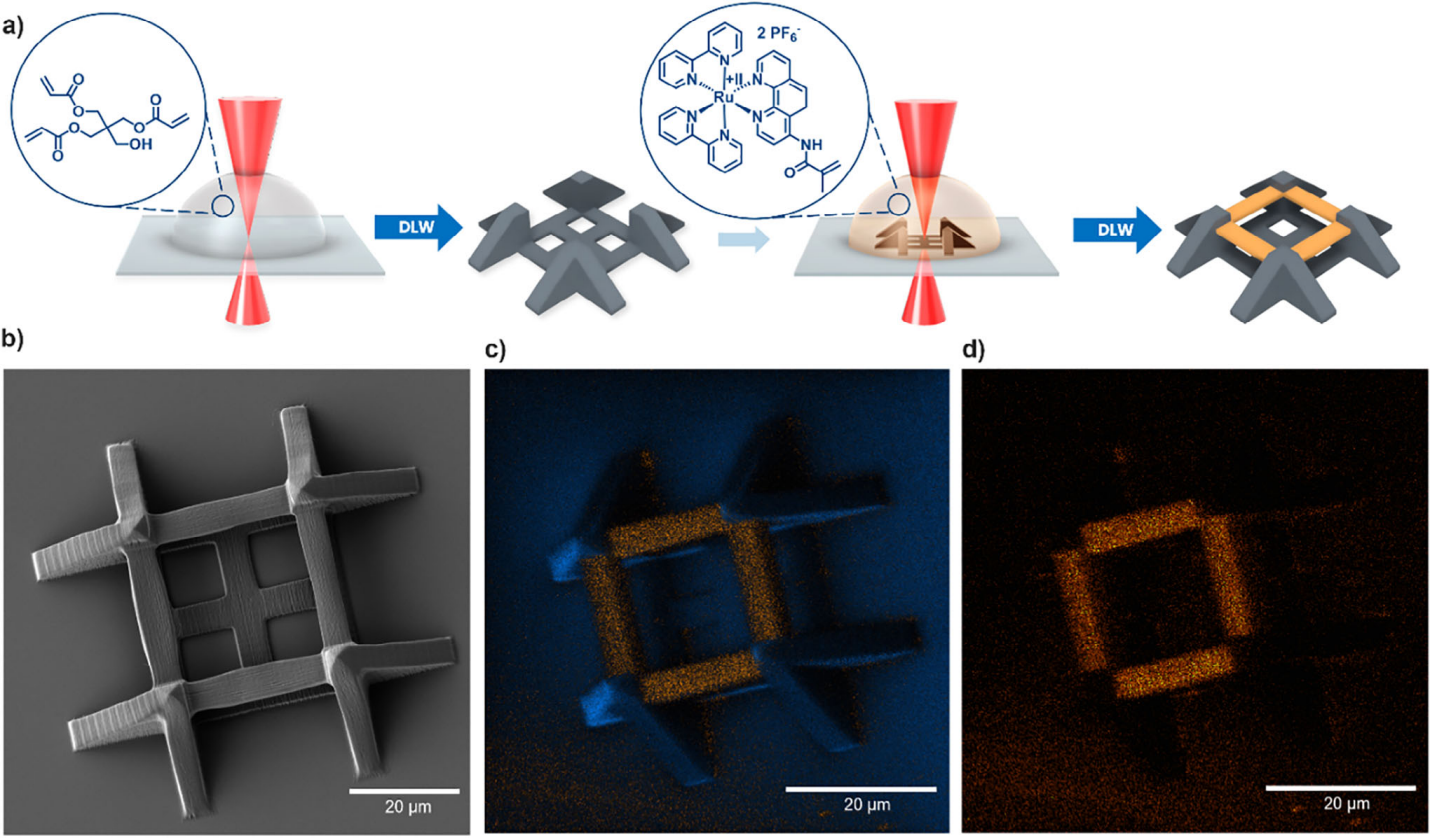
a) Fabrication of multi‐material boxing‐ring structures. Initially, DLW is used to print frames from a PETA‐based resist and in a second DLW step, the bridges are constructed from the **Ru(II)‐CM** resist (printed with a ratio of 20 wt.% **Ru(II)‐CM** to 80 wt.% PETA). b) SEM image of the multi‐material boxing‐ring print. c) ToF‐SIMS ion map, showing the overlaid intensities of fragments corresponding to Ru^+^ (orange) and fragments corresponding to C_3_H_5_^+^ (blue) of the polymer base. d) ToF‐SIMS ion map highlighting only the fragments corresponding to ruthenium (Ru^+^) ions.

Next, multi‐material scaffold‐type structures were fabricated to explore the spatial confinement of the catalyst within specific sections of the printed object. A common multi‐material design is a so‐called *boxing ring*, comprising four interconnected square pillars, which serves as a valuable example of a stable, freestanding 3D structure.^[^ ^25^ ^]^ A boxing ring design incorporates ribs to enhance substrate adhesion through increased contact area and improve structural stability. Fabrication of the boxing‐ring structure was conducted using 35 mW laser power and a scan speed of 10 000 µm s^−1^ in a two‐stage process. In the first step, the frames were printed using a PETA‐only resist formulation containing 2 wt.% of the initiator 4,4′‐bis(*N,N*‐diethylamino) benzophenone. Development of this print resulted in the base structure shown in **Figure** **3**a. Subsequently, bridges were printed from the **Ru(II)‐CM**‐containing resist (Figure 3a) and SEM of the dual‐material boxing ring was recorded (Figure 3b). The resulting microstructure was subjected to time‐of‐flight secondary‐ion mass spectrometry (ToF–SIMS) analysis to determine the spatial distribution of its chemical components. ToF–SIMS revealed that the presence of fragments with masses corresponding to ruthenium (Ru^+^) ions is confined to the bridges (Figure 3c,d, highlighted in orange), while such fragments are absent in the frame. These findings demonstrate the feasibility of precisely controlling the placement of catalytic material within intricately designed structures, thereby potentially enabling localized catalytic activity within structures.

**Figure 4:**
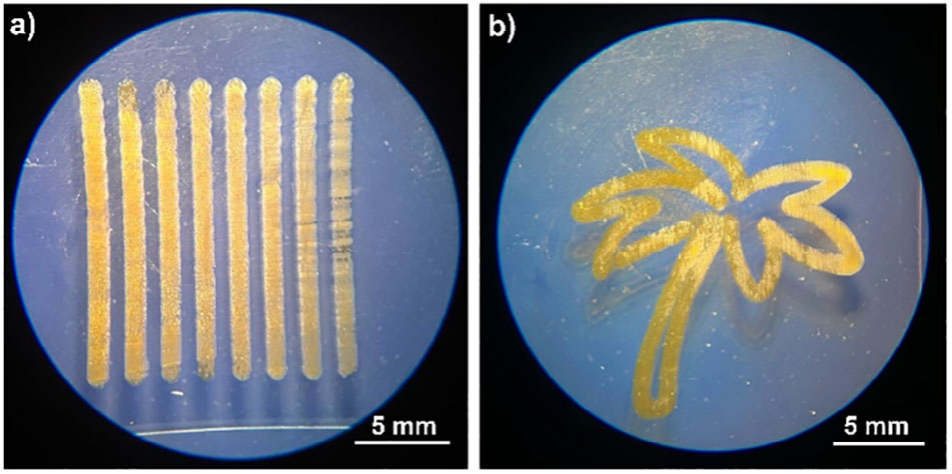
All structures shown were printed using a resist comprising 20 wt.% **Ru(II)‐CM** and 80 wt.% PETA and were fabricated using the Mono LISA. a) Printed line array with varying speeds, ranging from 0.10 to 0.45 in 0.05 mm s^−1^ increment. b) Palm tree printed with a velocity of 0.35 mm s^−1^.

After confirming the presence of Ru species within the printed structures, we investigated whether these are still photocatalytically active and accessible to substrates. To expedite the prototyping and testing of the catalytic activity of the printed structures and demonstrate the versatility of the developed resist system, we initially explored SLA printed structures. SLA was chosen due to its ability to swiftly produce larger structures, which facilitates initial catalytic evaluations. We employed the advanced 3D printer called the Monochromatic Tuneable Laser Integrated Stereolithographic Apparatus (Mono LISA), which was developed by our team.^[^ ^30^ ^]^ The Mono LISA setup fuses a monochromatic tunable laser system with a custom‐built 3D printing platform and enables printing at wavelengths between 210–2400 nm (refer to Supporting Information Section S2.6. for more details regarding the printer). The stage is controllable via customized software that leverages widely adopted G‐code files to precisely regulate both the printing path and velocity. To optimize the printing parameters, a series of line prints at varying speeds was conducted (**Figure** **4**a). The analysis revealed that a constant speed of 0.35 mm s^−1^ resulted in the most stable and precise scaffolds.

While the inherent resolution of one‐photon polymerization processes limits the direct comparison to two‐photon printing, we successfully fabricated well‐defined structures as depicted in Figure 4. The G‐code for the structures and their trajectories are provided in Supplementary files 2–4 and are graphically illustrated in Figure S7 (Supporting Information) respectively. The use of a consistent resin composition (20 wt.% **Ru(II)‐CM**, 80 wt.% PETA) for all structures demonstrated the versatility of the formulation across different writing techniques and scales.

**Scheme 2:**
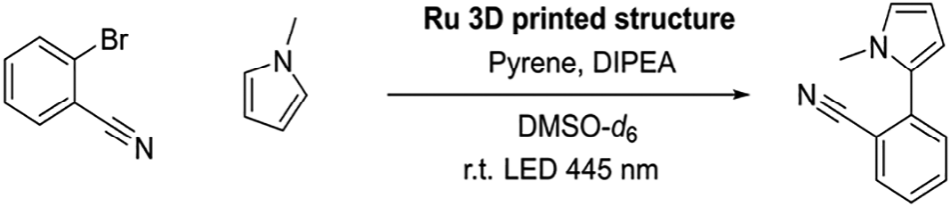
Photoredox C−H arylation of 2‐bromobenzonitrile. As a catalyst the 3D printed structures were used (fabricated both via DLW and SLA) (DIPEA=diisopropylethylamine).^[^ ^31^ ^]^

**Figure 5:**
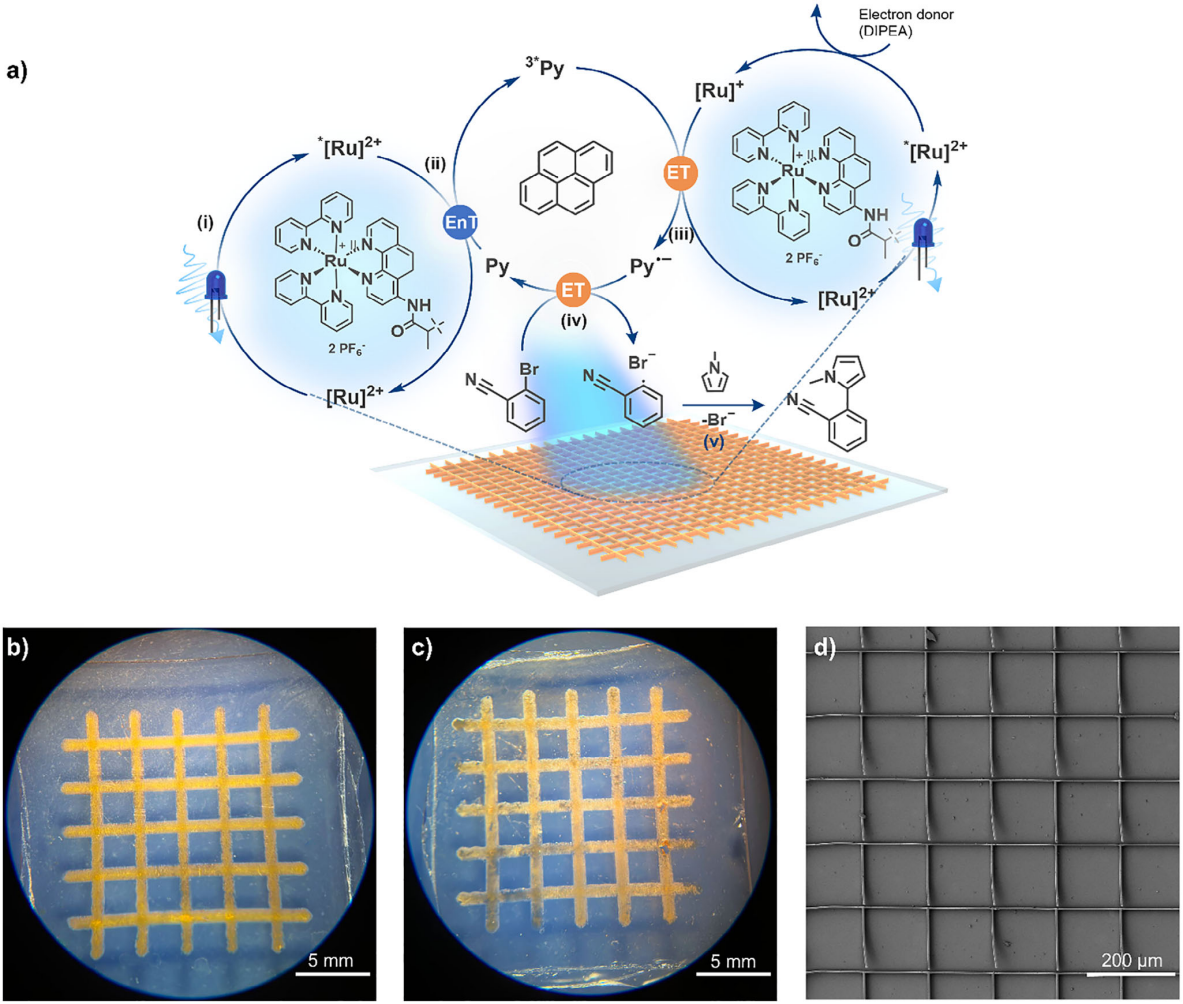
a) Adapted reaction mechanism of the C−H arylation of activated aryl bromides, elucidated by Moore.^[^ ^32^ ^]^ b) Photographs of the SLA printed mesh structure before the usage in the photocatalytic reaction and c) after the photocatalytic reaction. d) SEM picture of a section of the DLW‐written catalyst after the photocatalytic reaction.

Following the successful fabrication of larger catalyst‐containing structures using SLA printing, we investigated their catalytic activity (**Scheme** **2**). The Ru(II) photocatalyzed C–H arylation of activated aryl bromides^[^ ^31^ ^]^ was selected as a model reaction as it is reported to operate efficiently even at low catalyst loading, considering the fabrication time and size constraints of DLW. Extensive research has been conducted on the reaction mechanism, which enables C─C bond formation under mild conditions^[^ ^31^, ^32^, ^33^ ^]^ by utilizing two distinct light‐absorbing components to trigger a series of redox events. In a subsequent study, Moore and colleagues further investigated the reaction mechanism, proposing a five‐step pathway (**Figure** **5**a).^[^ ^32^ ^]^ Upon light irradiation, the Ru(II)‐complex undergoes immediate intersystem crossing i), which facilitates a triplet energy transfer, exciting the pyrene molecule ii). Subsequently, the excited pyrene is quenched by the reduced species of the Ru‐complex (Ru(I)), resulting in the formation of the pyrene radical anion (Py^•−^) iii). Subsequently, the pyrene radical anion initiates a halogen abstraction from 2‐bromobenzonitrile iv), resulting in its radical form. The 2‐bromobenzonitrile radical finally reacts with *N*‐methylpyrrole v).

The photocatalytic process usually involves [Ru(bpy)_3_]^2+^, which excites pyrene via energy transfer and subsequently reduces it. However, the printed structures incorporated a slightly different catalyst, as one bpy ligand was exchanged with *N*‐(1,10‐phenanthrolin‐5‐yl)methacrylamide. To investigate its catalytic behavior, we conducted a preliminary test using the monomeric **Ru(II)‐CM** in a small‐molecule study. This experiment utilized **Ru(II)‐CM** in powder form and contained 2‐bromobenzonitrile, *N*‐methylpyrrole, and diisopropylethylamine dispersed in DMSO‐*d*_6_. The mixture was deoxygenated and irradiation of the reaction solution with a 445 nm LED (refer to Supporting Information, Figure S1, Supporting Information) resulted in the consumption of 2‐bromobenzonitrile with a conversion of 20%, as evidenced by ^1^H NMR spectroscopy (Supporting Information: Figure S14 (Supporting Information), a detailed explanation of the calculation of the conversion can be found in Section S4.5).

**Figure 6:**
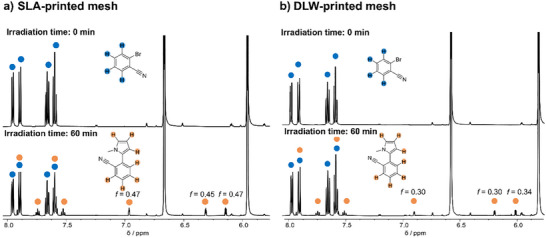
a) ^1^H NMR (600 MHz, DMSO‐*d*_6_) spectra of reaction solution using the SLA printed catalyst, before and after the irradiation time of 60 min. b) ^1^H NMR (600 MHz, DMSO‐*d*_6_) spectra of reaction solution using the DLW printed catalyst, before and after irradiation time of 60 min.

After the successful application of **Ru(II)‐CM** as a photocatalyst for C–H arylation, a mesh structure was printed for catalytic tests because of its high surface area (Figure 5b). The reaction with the SLA print was carried out under identical experimental conditions as the previously described monomeric **Ru(II)‐CM** test. After 60 min, ^1^H NMR spectra of the reaction solution revealed a conversion of 32% in the presence of the SLA‐printed catalyst (**Figure** **6**a). After the catalysis, the structure of the mesh remained largely intact (Figure 5c). A direct comparison to the catalytic activity of the ruthenium complex in the small molecule study is challenging as only the surface area of the 3D‐printed structures is accessible for catalysis. Further, due to the novelty of our approach, direct comparability to existing ruthenium‐based printed photocatalysts is very limited. The conversion is similar to previously reported studies.^[^ ^34^ ^]^

Building upon the successful catalysis of the SLA print, a smaller and finer mesh structure was printed via DLW (Figure 5d). Using a 65 mW laser power and a scan speed of 125 000 µm s^−1^, the entire structure took close to 8 h to print and was completed in multiple sessions and used the same resist as the SLA printed catalyst. The DLW‐mesh structure measures an area of ≈3 × 2 mm, significantly smaller than the 16 × 16 mm dimensions of the SLA‐printed structure. The DLW‐printed object was subjected to identical photochemical reaction conditions as the SLA‐print and the small‐molecule study (Supporting Information Section 4.3–4.5.). Despite the reduced scale, our DLW‐printed structure exhibited effective catalytic activity, achieving a conversion of 24% after 60 min of irradiation. Subsequently, we explored the performance of the printed mesh in several cycles for catalysis using a fresh reaction solution. The repeat catalysis – using a completely newly yet identically DLW fabricated mesh – demonstrates a near constant conversion (between close to 28% and 23%) and thus a continuously high performance of the catalytic two‐photon printed structure (refer to the Supporting Information Section S4.6, Table S4, for details of the repeat catalysis). The DLW‐printed mesh exhibits a conversion that is only 75% of the conversion of the SLA‐printed mesh (24% vs 32%, **Table** **1**). Importantly, however, the volume of the DLW mesh is ≈1% of the volume of the SLA‐printed mesh (1.239 vs 0.0153 mm^3^) and the surface‐to‐volume ratio of the DLW‐printed mesh is five times higher than the SLA‐printed mesh.

**Table 1:** Comparison of the photocatalytic activity of the SLA‐ and DLW‐printed Ru‐based mesh in the C─H arylation of activated aryl bromides (cf. Figure 5). Refer to Supporting Information Sections S4.3–S4.6 for experimental details.

Type	Volume [mm^3^]	Surface [mm^2^]	Surface to Volume Ratio [mm^−1^]	Conversion [%]
SLA‐printed mesh	1.239	123	99.27	31.6
DLW‐printed mesh	0.0153	7.8	509.8	23.9

Using two orders of magnitude less catalyst and overall 3D printed material leads to a higher surface‐to‐volume ratio and enables a catalytic performance within the same range of catalytic efficiency of the larger SLA mesh structure. Future work will focus on investigating the beneficial geometry of the DLW mesh. These results demonstrate the significant potential of DLW‐printed catalysts for advancing sustainable fabrication of both materials and chemicals and expand on the opportunities offered by conventional catalyst immobilization techniques in the context of ruthenium polymers.^[^ ^35^ ^]^ Considering the controlled placement of catalytic material achieved in the current study, our approach holds significant potential for the fabrication of flow reactors. We envisage investigating methods for accurately quantifying the catalytically active species during the reaction and improving efficiency by exploring different printed geometries and varying porosity.

## 3 Conclusion

We introduce a photoresist system that enables the direct placement of catalytically active sites within intricate 3D printed geometries, resulting in the first example of a light‐induced 3D printed micro‐ and macro‐catalytic system from a dual function photoresist. Our system leverages a **Ru(II)‐CM** functionalized with a polymerizable moiety, allowing for the assembly of 3D objects using commercially available crosslinkers. Notably, the photoinitiation process also occurs via the Ru(II)‐complex, which gives rise to the photocatalytic activity of the printed object. We demonstrate the fabrication of multi‐material microstructures and confirm the presence of ruthenium on the surface of these structures via ToF‐SIMS. Our approach thus enables the direct utilization of the printed structures as catalysts as demonstrated on mesh structures fabricated using both one‐ and two‐photon techniques.

Their catalytic activity was exemplified through the C─H arylation of activated aryl bromides. Based on this reaction, the effect of the printed design on its catalytic activity was demonstrated. A microscale mesh structure yielded a photocatalytic performance comparable to a macroscale mesh, despite requiring only 1% of its volume. Ultimately, our approach holds significant potential for the fabrication of complex catalytically active devices on demand.

## Conflict of Interest

The authors declare no conflict of interest.

## Supporting information



Supporting Information

Supporting Information

## Data Availability

The data that support the findings of this study are available in the supplementary material of this article.
