# Supplementary material for: Catalytically Active Light Printed Microstructures
Source: Adv Mater. 2025 Jun 6;37(34):2506663. doi: 10.1002/adma.202506663 (PMC12392858; doi:10.1002/adma.202506663)
Supplement: Supplementary file 1 — Supporting Information [file ADMA-37-2506663-s001.pdf]

ADVANCED MATERIALS

Supporting Information

for *Adv. Mater.*, DOI 10.1002/adma.202506663

Catalytically Active Light Printed Microstructures

Alicia K. Finch, Sebastian Gillhuber, Hendrik Frisch, Peter W. Roesky* and Christopher Barner-Kowollik**

Supporting Information

Catalytically Active Light Printed Microstructures

Alicia K. Finch, Sebastian Gillhuber, Hendrik Frisch,* Peter W. Roesky,* Christopher Barner-Kowollik*

Contents

1 Materials and Methods S3
2 Analytical Techniques S4
2.1 Nuclear Magnetic Resonance Spectroscopy S4
2.2 Liquid Chromatography Mass Spectrometry S4
2.3 DMAC-Size Exclusion Chromatography S4
2.4 LED Characterization S5
2.5 Direct Laser Writing S5
2.6 Monochromatic Tuneable Laser Integrated Stereolithographic Apparatus (Mono LISA) S10
2.7 ToF-SIMS Measurements S12
2.8 Scanning Electron Microscopy S13
3 Synthetic Procedures S13
3.1 Synthesis of Ruthenium-Monomer S13
3.2 Synthesis of 5-nitro-1,10-phenanthroline (**1**) S13
3.2 Synthesis of 5-amino-1,10-phenanthroline (**2**) S14
3.3 Synthesis of *N*-(1,10-phenanthrolin-5-yl) methacrylamide (**3**) S15
3.4 Synthesis of bis(2,2′-bipyridine)-(1,10-phenanthrolin-5-yl-methacrylamide)ruthenium(II) bis(hexafluorophosphate) (**Ru(II)-CM**) (**4**) S16
4.1 Irradiation set-up used for catalytic experiments S17
4.2 Printed structures for catalytic experiments S18
4.3 Molecular Catalysis S19
4.4 Catalysis with 3D Printed Mesh Structures S20
4.5 Calculation of Conversion S22
4.6 Recycling Catalysis Studies with DLW Printed Mesh S23
4.7 Calculation of the Surface of both the SLA and DLW Mesh S24
5 Analytical Data S27
5.1 Nuclear Magnetic Resonance (NMR) Spectra S27
5.2 Mass Spectrometry S29
5.3 Additional ToF-SIMS Images S31
References S31

1 Materials and Methods

All chemicals and solvents were used as received from the supplier without further purification, unless stated otherwise.

Acetone (HPLC grade, Thermo Fisher), acetonitrile (Chemsupply), tetrahydrofuran (Thermo Fisher), ethyl acetate (Thermo Fisher), dichloromethane (Chemsupply), methanol (Thermo Fisher), dimethyl sulfoxide (Thermo Fisher), dimethylformamide (Thermo Fisher), ethanol (Thermo Fisher), water (Milli-Q, Merck), toluene (Thermo Fischer), propylene carbonate (Sigma Aldrich), 3-(trimethoxysilyl) propyl methacrylate (Sigma Aldrich), pentaerythritol triacrylate (PETA, Sigma Aldrich), sodium bicarbonate (Sigma-Aldrich), hydrochloric acid (Thermo Fisher), sulfuric acid (Thermo Fisher), magnesium sulfate (Merck), ruthenium(III) chloride (Combi-Blocks), lithium chloride (99%, Sigma-Aldrich), 1,10-phenanthroline, (99% Sigma-Aldrich), triethylamine (99.5%, Thermo Fisher), methacryloyl chloride (97%, Merck), 2-bromobenzonitrile (99%, Merck), *N*,*N*-diisopropylethylamine (DIPEA, 99.5%, Sigma Aldrich), ammonium hexafluorophosphate (97%, Thermo Fisher), palladium on carbon (5 wt. % loading, Sigma Aldrich). Chloroform-d_1 ($CDCl_3$, 99.8% D, Sigma-Aldrich) and dimethyl sulfoxide-d_6 (DMSO-d_6), 99.9% D, Sigma-Aldrich) were utilized as solvents for NMR measurements. All chemicals were used as received unless stated otherwise. *N*-Methylpyrrole was passed through a neutral alumina column for purification. Water was purified by an SP-1 MilliQ purification system, and dichloromethane was purified by a solvent purification system.

2 Analytical Techniques

2.1 Nuclear Magnetic Resonance Spectroscopy

^{1}H NMR and ^{13}C NMR spectra were recorded on a Bruker System 600 Ascend LH, equipped with a BBO-Probe (5 mm) with z-gradient (^{1}H: 600.13 MHz, ^{13}C 150.90 MHz). The δ-scale was normalized relative to the solvent signal of $CHCl_3$ or DMSO for ^{1}H spectra and for ^{13}C spectra on the middle signal of the $CHCl_3$ triplet and the DMSO quintet. The abbreviations for the couplings were given as: s: singlet, d: doublet, t: triplet, q: quartet, m: multiplet.

2.2 Liquid Chromatography Mass Spectrometry

LC-MS measurements were performed on an Dionex UltiMate 3000 UHPLC system consisting of a quaternary pump (LPG 3400RS), autosampler (WPS 3000TRS) and column oven (TCC 3000). A 10 µL aliquot of sample was injected onto a C18 HPLC column (Phenomenex Luna 5 µm, 100 Å, 250 × 2.0 mm) maintained at 40 °C. Mobile phase A was 5 mM ammonium acetate in water and mobile phase B was acetonitrile. The combined flow rate was maintained at 0.4 mL/min. After an initial 0.6 min isocratic period at 20 % B, the gradient was increased to 95 % B over 7 min then held at 95 % B for a further 3 min before returning to 20 % B. The eluate was directed to a UV diode array detector (DAD 3000, Dionex) and subsequently into the heated electrospray ionisation source of a Q Exactive Plus Biopharma high-resolution Orbitrap mass spectrometer (Thermo Fisher Scientific, Bremen, Germany). The spray voltage was set to 3.0 kV, and the sheath and auxiliary gas (N_2) flow rates were set to 30 and 10 (dimensionless arbitrary units), respectively. The capillary temperature was set to 320 °C, the S-lens RF level was set to 60, and the auxiliary gas heater temperature was set to 150 °C. Spectra were acquired at a nominal mass resolving power of 70,000 (defined at *m/z* 200).

2.3 DMAC-Size Exclusion Chromatography

The SEC measurements were conducted on a *PSS* SECurity^2 system consisting of a *PSS* SECurity Degasser, *PSS* SECurity TCC6000 Column Oven (60 °C), *PSS* GRAM Column Set (8x150 mm 10 µm Precolumn, 8x300 mm 10 µm Analytical Columns, 1000 Å, 1000 Å and 30 Å) and an *Agilent* 1260 Infinity Isocratic Pump, *Agilent* 1260 Infinity Standard Autosampler, *Agilent* 1260 Infinity Diode Array and Multiple Wavelength Detector (A: 254 nm, B: 360 nm), *Agilent* 1260 Infinity Refractive Index Detector (35 °C). HPLC grade DMAc, 0.01 M LiBr, is used as eluent at a flow rate of 1 $mL \cdot min^{-1}$. Narrow disperse linear poly(styrene) (M_n: 266 $g \cdot mol^{-1}$ to 2.52×10^6 $g \cdot mol^{-1}$) and poly(methyl methacrylate) (M_n: 202 $g \cdot mol^{-1}$ to 2.2×10^6 $g \cdot mol^{-1}$) standards (*PSS* ReadyCal) were used as calibrants. All samples were passed over 0.22 µm PTFE membrane filters. Molecular weight and dispersity analysis was performed in *PSS* WinGPC UniChrom software (version 8.2).

2.4 LED Characterization

LED emission spectra were recorded using an Ocean Insight Flame-T-UV-Vis spectrometer, with an active range of 200-850 nm and an integration time of 10 ms. LED output energies were recorded using a Thorlabs S401C thermopile sensor, with an active area of 100 mm^2 and a wavelength range of 190 nm – 20 µm, connected to a Thorlabs PM400 energy meter console. The emitted power from each LED was measured for 60 s at a fixed distance from the sensor, after which the mean and standard deviation of the emission could be determined. LEDs were cooled during measurement to minimize any thermal effects on the emission power or sensor performance.

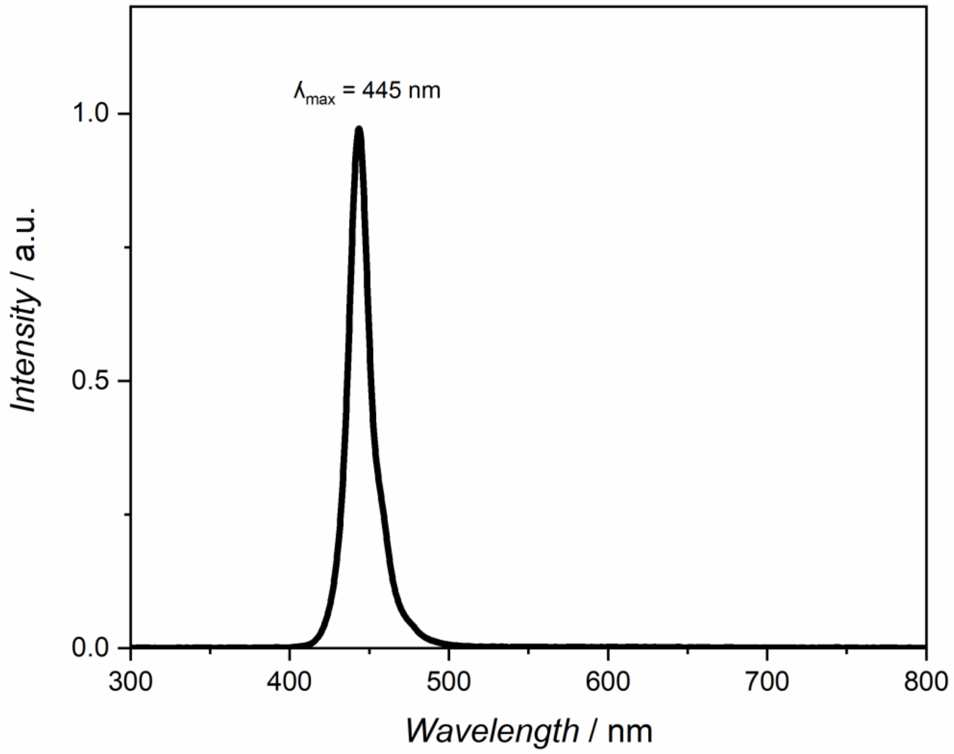

Figure S1. Emission profile for the 445 nm 10 W LED.

2.5 Direct Laser Writing

DLW was performed with a commercially available DLW system (Photonic Professional, Nanoscribe GmbH) equipped with a 100× NA (numerical aperture) = 1.4 oil-immersion objective. Typical average laser powers in the DLW process were 20–40 mW (in front of the microscope lens) and the typical galvo scanning velocity was between 2000 and 20000 µm s^{-1}. Prior to writing, all glass substrates were functionalized with 3-(trimethoxysilyl) propyl methacrylate to enhance the adhesion of the microscaffolds. The glass substrates were plasma cleaned for 2 h, followed by immersion in a 5 mM solution of 3-(trimethoxysilyl) propyl methacrylate in toluene for 1 h. Subsequently, the substrates were rinsed with toluene as well as acetone and

dried nitrogen. The microstructures written from different resist compositions are presented in the following.

Table S1. Compositions of the photoresists used to write the structures within the current study. All propylene carbonate is assumed to have been washed out through the described washing procedure. All acrylate species are assumed to react with equal efficiency, leading to the given ratios in the structures. The photoinitiator 4,4'-bis(*N,N*-diethylamino) benzophenone (DEAPB) was used for the printing of the functionalized base structure of the boxing ring.

Photoresist	Ru(II)-CM [mg]	PETA [mg]	PI (DEABP) [mg]
**P1** (20 wt% **Ru(II)-CM** / 80 wt% PETA)	62.5	250	-
PETA -only	/	250	5.1

The microstructures were all written from the photo-responsive resist **P1** with laser powers ranging between 20 mW and 65 mW and the scan speed was varied between 8000 µm s^{-1} and 125000 µm s^{-1}, depending on the printed structure. An overview of each structure is provided in Table S2. Optimal writing conditions were determined by varying laser power and writing speed during printing tests (Figure S2 and S3).

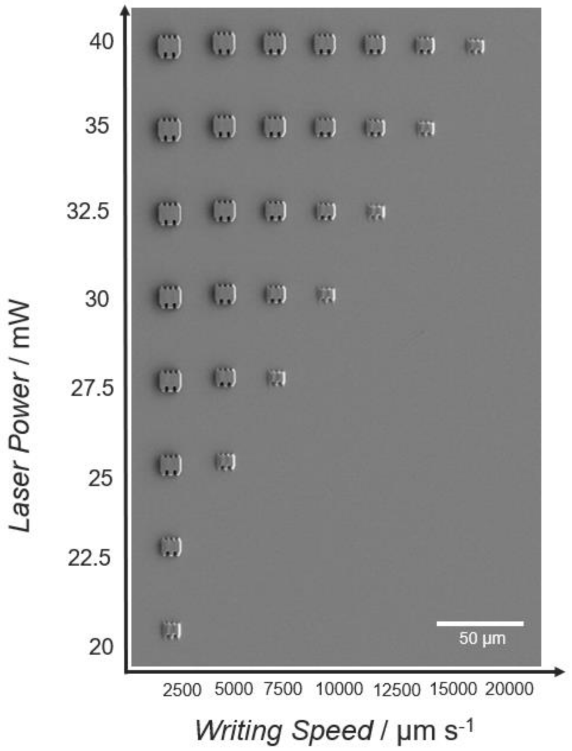

Figure S2. SEM image of direct laser written 3D spiky blocks (14.5 x 13.5 x 5 µm) at different laser powers and writing speeds. Axes indicate the laser powers and scan speeds the structures were written with. Printed from resin **P1**.

Through printing tests, we can compare photoinitiation efficiency to other commercial resists. Herein, we conducted printing test with the resin containing 20 wt% of the ruthenium-monomer. Using a dimensionless Figure-of-Merit (FOM) applicable across all experiments on a given photoresist, we can compare the sensitivity of different photoresists. A higher FOM indicates greater photoresist sensitivity.[1] The following equation was used to determine the FOM:

$$\mathrm{FOM} = vP_{\mathrm{th}}^{-2}\lambda_0^3 R_{\mathrm{p}} t_{\mathrm{p}} (\mathrm{NA})^{-3}$$

Table S2. Symbols, Parameters and Units used in the dimensionless Figure-of-Merit.

Symbol	Parameter	unit
v	scan speed	m/s
P_{th}	threshold laser power	W
λ_0	Free-space wavelength	m
R_p	pulse repetition rate	Hz
t_p	pulse length	s
NA	numerical aperture of focus lens	-

The values in Table S3 were determined for the calculation of the FOM, here the SEM picture of the print in Figure S2 was used to obtain values for writing speed and laser power limits. The obtained FOM of 53 for our system is comparable to the FOM of a mixture of Irgacure 369 and PETA (56).

Table S3. Values, which were used to calculate the FOM.

Initiator	Monom.	v [µm s^{-1}]	LP [%]	P_{th} [W]	λ_0 [nm]	R_p [MHz]	t_p [fs]	NA	FOM
Ruthenium-Monomer	PETA	20000	40	0.0720	780	80	100	1.4	53

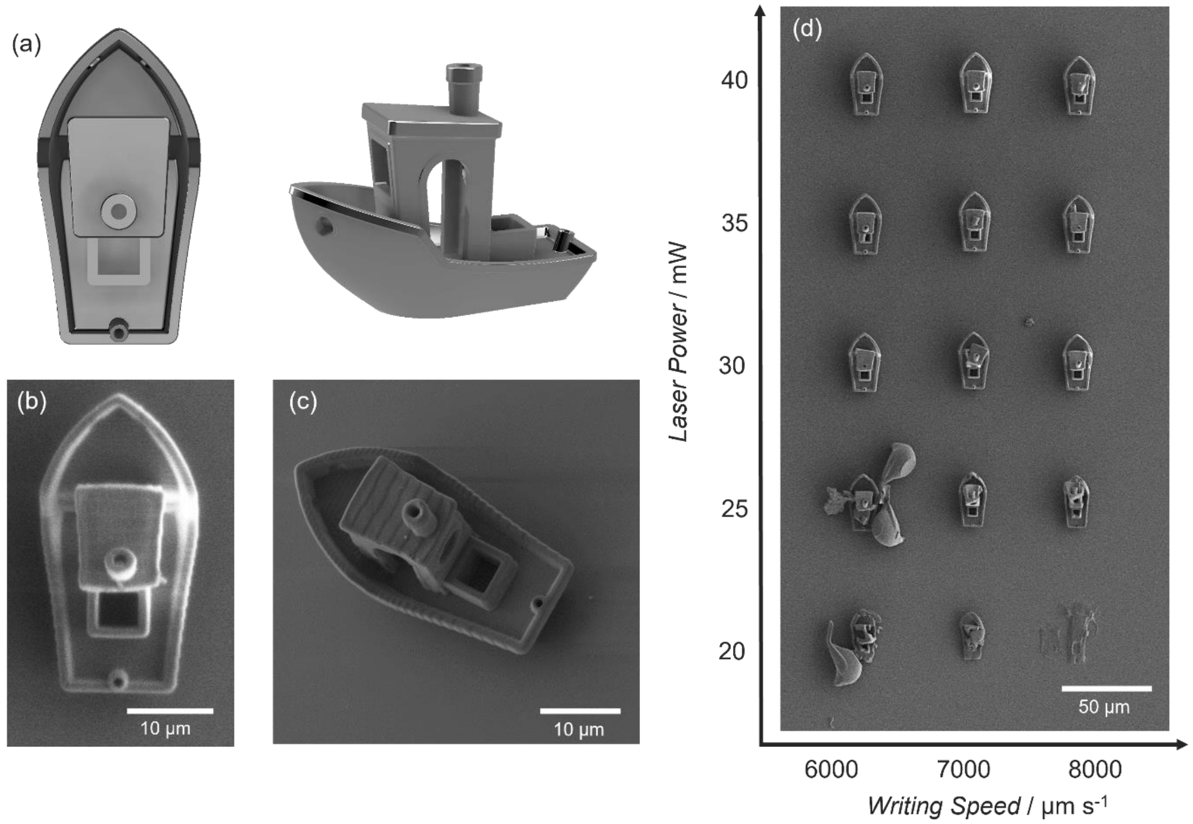

Figure S3. (a) Digital model of the boat (b)-(c) SEM images of direct laser written boats written at 6000 µm s^{-1} and a laser power of 35 mW. (d) SEM image of direct laser written boats at different laser powers and writing speeds to obtain the best writing conditions for this specific structure. All structures were printed with the resin containing a ratio of 20 wt% Ru(II)- complex to 80 wt% PETA.

Table S4. All DLW printed structures and their printing conditions.

Structure	Laser Power [mW]	Scan Speed [µm s^{-1}]	Resin
Sunflower	35	10000 µm s^{-1}	P1
Butterfly	35	10000 µm s^{-1}	P1
Boat	35	6000 µm s^{-1}	P1
Boxing Ring Base	35	10000 µm s^{-1}	PETA only
Boxing-Ring Bridges	35	10000 µm s^{-1}	P1
Mesh-Catalyst	65	125000 µm s^{-1}	P1

The boxing ring structures were written in two steps. In the first step, the frames were written from a PETA/DEABP resist (laser power = 20 mW, scan speed = 10000 µm s^{-1}). After the first writing step, the samples were developed in the way described below. In a second step, the bridges between the frames were written from the photo-

responsive resist **P1** and developed in the same way afterwards. After writing, all samples were developed by immersing the cover slips in acetone for at least 10 min.

Further, we conducted a study to determine the highest concentration of **Ru(II)-CM** incorporated into the resin. Initial prints were conducted with 10 wt% of **Ru(II)-CM** (**P0**), an example can be seen in Figure S4, then the concentration was increased as per Table S3. Printing was possible up to 20 wt% of **Ru(II)-CM**. Increasing the concentration further, made the printing not possible due to laser penetration issues.

Table S5. Compositions of the photoresists used to determine the maximum loading of the complex to direct laser write 3D microstructures. 10 µL propylene carbonate was added to each resin mixture to ensure the components were dissolved. Printing was possible up to 20 wt% of **Ru(II)-CM** (S2), a result for 10 % can be seen in S4.

Photoresist	Ru(II)-CM [mg]	PETA [mg]	Complex to PETA [wt%]
P0	28.4	250	10/90
P1	62.5	250	20/80
P2	83.3	250	25/75
P3	107.1	250	30/70
P4	166.7	250	40/60

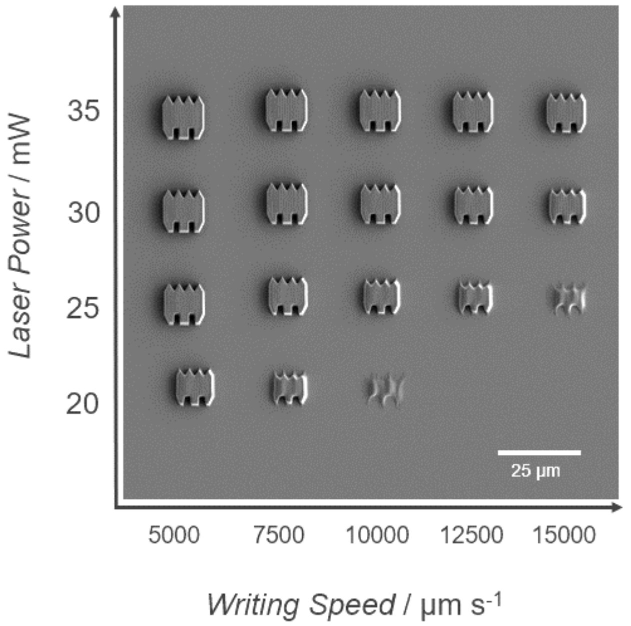

Figure S4. SEM image of direct laser written 3D spiky blocks (14.5 x 13.5 x 5 µm) at different laser powers and writing speeds. Axes indicate the laser powers and scan speeds the structures were written with. Here, microstructures were written from the **P0**-based photoresist.

Further, the effect of functionalizing the ruthenium complex on the resulting print was examined. Here, two different resists were printed, **P1** was used with a 20 wt% of the Ru(II)-CM (see Table S3), as well as a resist containing tris(bipyridine)ruthenium(II) hexafluorophosphate instead of Ru(II)-CM. Identical formulations were used, however

the tris(bipyridine)ruthenium(II) hexafluorophosphate did not dissolve as well as the Ru(II)-CM in the PETA and propylene carbonate mixture, resulting in dispersed crystals, visible during the printing process, as illustrated in Figure S5. Post-printing, distinct differences were evident. Developing in acetone resulted in holes within the structure printed with the non-functionalized complex, suggesting that the catalyst dissolved and was not incorporated into the polymer matrix during printing. Conversely, the print of the functionalized complex showed a uniform color distribution throughout the whole structure.

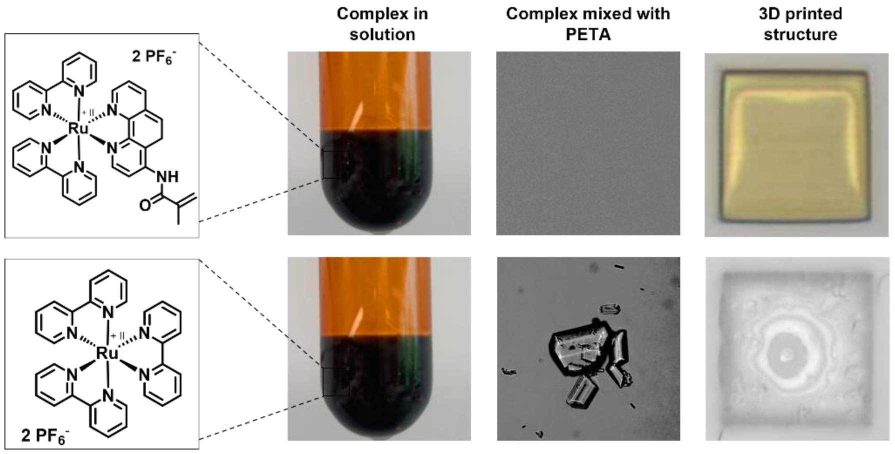

Figure S5. The top row illustrates results with resist **P1**, where the incorporation of Ru(II)-CM facilitated good dissolution of the PETA-based resin, observable under the microscope during printing. Subsequent development yielded a uniformly yellow print. Conversely, when **Ru(II)-CM** was replaced with tris(bipyridine)ruthenium(II) hexafluorophosphate, the resulting resin contained dispersed crystals, leading to a final print exhibiting holes where the tris(bipyridine)ruthenium(II) hexafluorophosphate had dissolved.

2.6 Monochromatic Tuneable Laser Integrated Stereolithographic Apparatus (Mono LISA)[2]

All components of the 3D printing system are mounted on an optical table. As shown in Scheme S1, an Opotek Opolette 355 OPO wavelength-tunable laser producing 7 ns, 20 Hz pulses with a flattop signal profile (①) serves as the light source. The output beam reflects through UV-enhanced aluminum mirrors (③ and ④, avg. reflectance > 90%) and subsequently passes through an electronic shutter (⑤). It further passes a spherical focusing lens (⑥, f = 300 mm) mounted on a long-travel stage and directs to a 3-axis stage (⑧, Thorlabs motorized translation stage PT3-Z29 with three K-Cube DC Servo Motor Controllers) using a UV silica right angle prism (⑦, 185 nm - 2.1 μm). The power meter (②) is positioned between the laser and the first mirror to measure all powers for printing. The resin tank is placed under the X, Y and Z stage by a tank holder and the build platform with a movable bar is mounted on the X, Y and Z stage. Photographs of the printing platform including the 3-axis (X, Y and Z) stage, build platform, resin tank and tank holder as well as models of these three components, are shown in Figure S1. The movement of the long-travel stage, the X, Y and Z stage as well as the shutter (on-off) is controlled by our customized software

(Supplementary file 1), programmed in LabView. Upon importing G-code files containing the coordinates and travel speed (Supplementary files 2-5), the software processes the files to precisely control both the printing trajectory and speed.

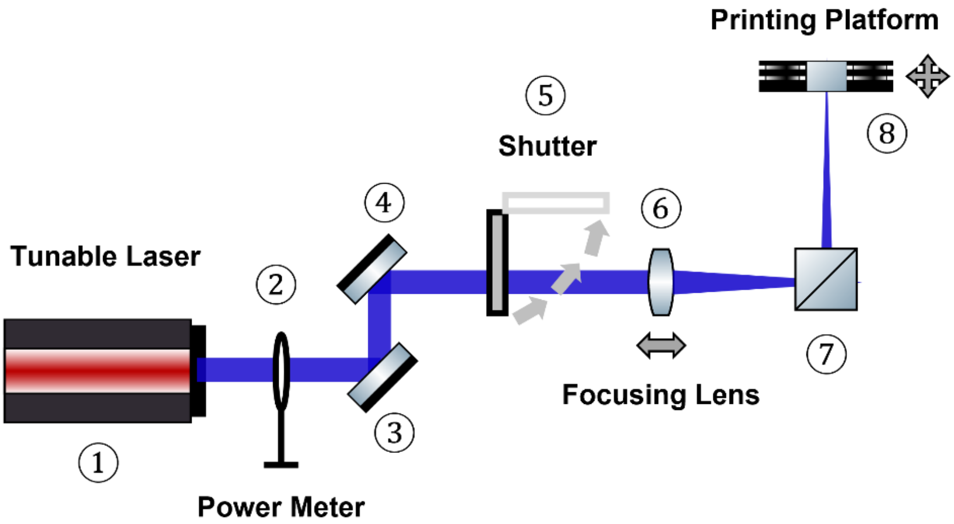

Scheme S1. Schematic of the Mono LISA printer, showing the beam path and various components.[2]

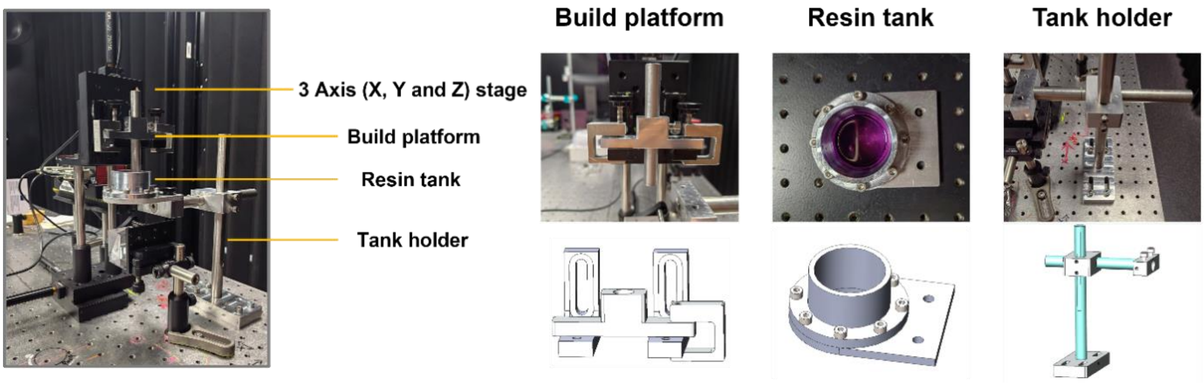

Figure S6. Photographs of the wavelength-tunable laser printing platform and the CAD models of three components.[2]

The LabView software is used to establish communication with each of the motors' controllers of the 3-axis (X, Y and Z) stage and the long-travel stage. Once communication is established, LabView can automate sending the X, Y and Z coordinates to the controllers of the 3-axis stage, allowing the three motors to move at the same time, as well as defining the velocity for each axis. Furthermore, to automate a series of commands to enable 2D and 3D printing, the LabView program is written to accept the widely used G-code file format. G-code files can be generated using commercially available software, such as UltiMaker Cura (v. 5.6.0).

The SLA printing process utilized photoresin **P1** containing 20 wt% Ru(II)-monomer (**Ru(II)-CM**), identical to that employed in the previous DLW experiments. This resin was prepared by mixing 1000 mg of PETA with 250 mg of **Ru(II)-CM** and adding 4 wt% propylene carbonate to ensure complete dissolution of all components (10 wt% with

respect to 1250 mg of resin). Following the placement of the glass sample holder on the X, Y, and Z stage, the photoresist was deposited onto the glass slide. G-code files for the printed structures (refer to Figure S6) were generated using UltiMaker Cura (v. 5.6.0) and Autodesk Fusion (2025). The laser beam was focused onto the glass substrate by adjusting the long-travel stage. Subsequently, the stage was moved in the X and Y directions under software control to print the desired objects. The printing speed for the line structure varied between 0.1 to 0.5 mm s^{-1} and was increased in 0.05 mm s^{-1} increments. The palm tree and butterfly were printed with a speed of 0.35 mm s^{-1}. The laser power used for all prints was 0.47 ± 0.025 mW at 390 nm. After laser exposure, all printed structures were developed by immersion in acetone. Supplementary Files 2-5 provide the G-code files for all structures. Figure S6 illustrates the trajectories and coordinates defined within these G-code files for each structure.

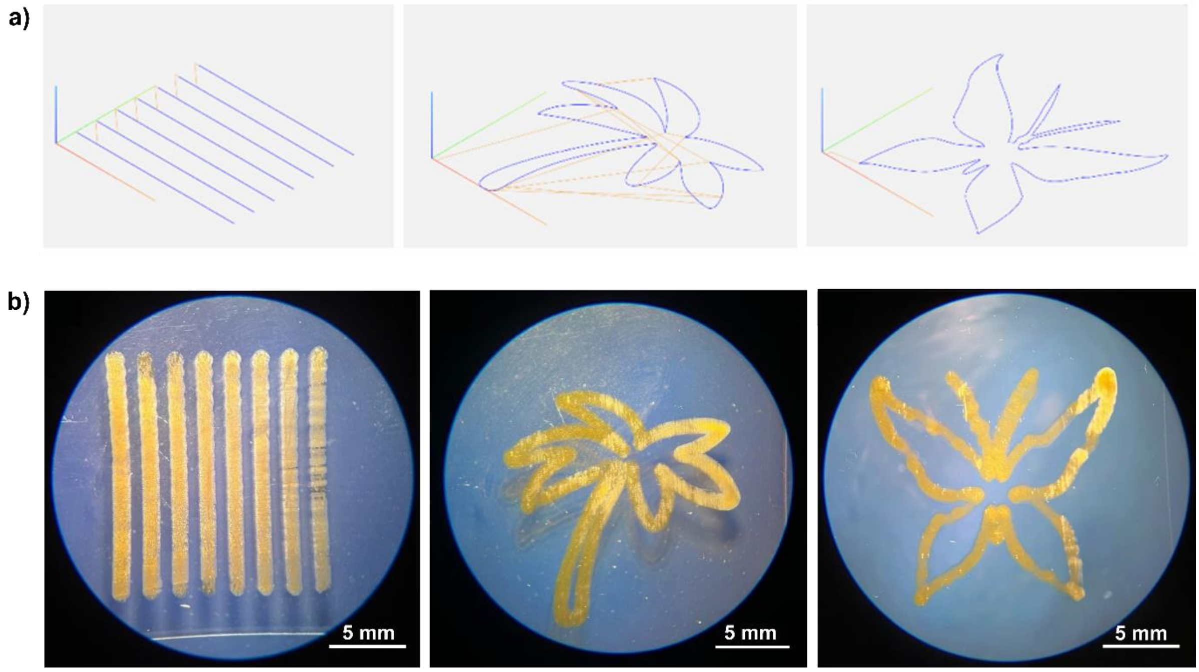

Figure S7. (a) (X, Y, and Z) stage trajectories for the printed lines, palm tree, and butterfly structures, generated from the G-code files provided in Supplementary Files 2-4. Blue lines represent the trajectory during laser exposure (shutter open), while orange lines indicate movement with the laser shutter closed. (b) Printed structures contained within the codes.

2.7 ToF-SIMS Measurements

ToF-SIMS data were acquired using an IONTOF M6 instrument (IONTOF GmbH, Münster, Germany) equipped with a reflectron time-of-flight analyser and Bi/Mn primary-ion source. Bi_3^+ cluster ions were selected from the pulsed primary-ion beam for the analysis. In order to attain the sub-micron spatial resolution required to image the scaffolds whilst also maintaining good mass resolution ($m/\Delta m$ > 5000), the pulses

were ‘unbunched’ and the analyser operated in delayed-extraction mode. To compensate for surface charging, the sample was flooded with low-energy (21 eV) electrons between primary-ion pulses. Spectra were acquired in positive polarity, and the mass scale calibrated using peaks attributed to hydrocarbon ions ($C_2H_3^+$, $C_3H_3^+$, $C_4H_3^+$, $C_5H_3^+$, $C_6H_3^+$). During data acquisition, the pressure in the analysis chamber was maintained at, or below, 1×10^{-8} mbar.

2.8 Scanning Electron Microscopy

SEM images were captured using a Tescan MIRA3 at 10 kV equipped with a secondary electron detector. Each sample was coated with either a 10 nm layer of gold or 2 nm layer of platinum.

3 Synthetic Procedures

3.1 Synthesis of Ruthenium-Monomer

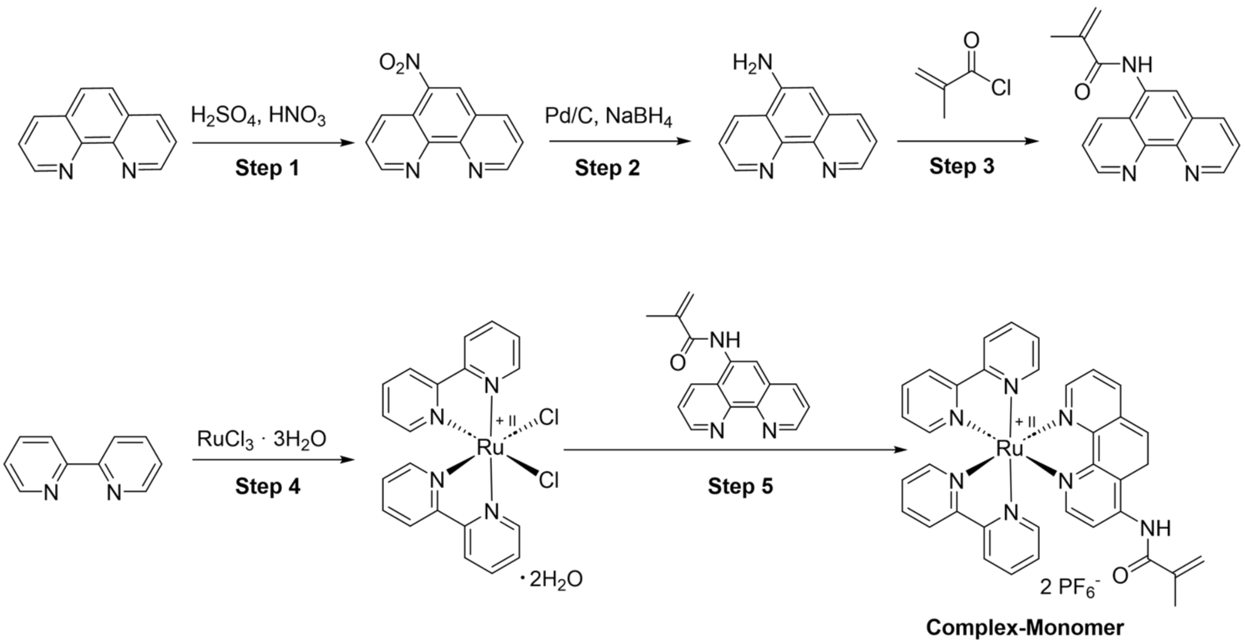

Scheme S2. Synthesis of the ruthenium-monomer (**Ru(II)-CM**).

3.2 Synthesis of 5-nitro-1,10-phenanthroline (1)

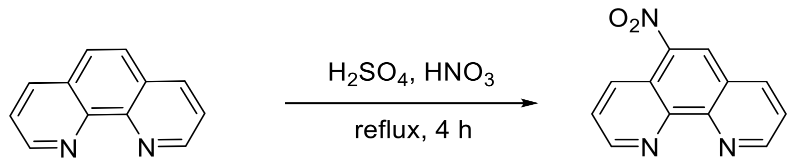

(1)

5-Nitro-1,10-phenanthroline was synthesised as previously described with minor synthetic modifications.[3] 1,10-Phenanthroline (1.00 g, 22.19 mmol) was dissolved in an ice-cold mixture of concentrated sulfuric acid and fuming nitric acid (2:1 v/v, 15 mL). Subsequently, the mixture was refluxed for 3 h at 160 °C and subsequently poured onto ice. The pH of the resulting solution was adjusted to 3 using an aqueous solution

of sodium hydroxide, leading to the precipitation of a pale-yellow solid. The solid product was isolated by vacuum filtration and washed with water and dried under reduced pressure. **Yield:** 1.11 g, 89%.

^{1}H NMR (600 MHz, $CDCl_3$, 298 K): *δ* (ppm) = 9.29 (dd, *J* = 4.3, 1.8 Hz, 1H), 9.25 (dd, *J* = 4.2, 1.6 Hz, 1H), 9.05 (s, 1H), 8.90 (dd, *J* = 8.6, 1.6 Hz, 1H), 8.79 (dd, *J* = 8.1, 1.8 Hz, 1H), 7.95 (ddd, *J* = 17.5, 8.3, 4.3 Hz, 2H).**^{13}C NMR (150 MHz, DMSO, 298 K):** *δ* (ppm) = 120.8, 124.9, 125.0, 126.0, 126.4, 132.6, 138.9, 140.2, 146.0, 147.3, 151.7, 153.9. **LC-MS:** calculated: *m/z* = 226.0611 $[M+H]^+$; found: *m/z* = 226.0612 $[M+H]^+$.

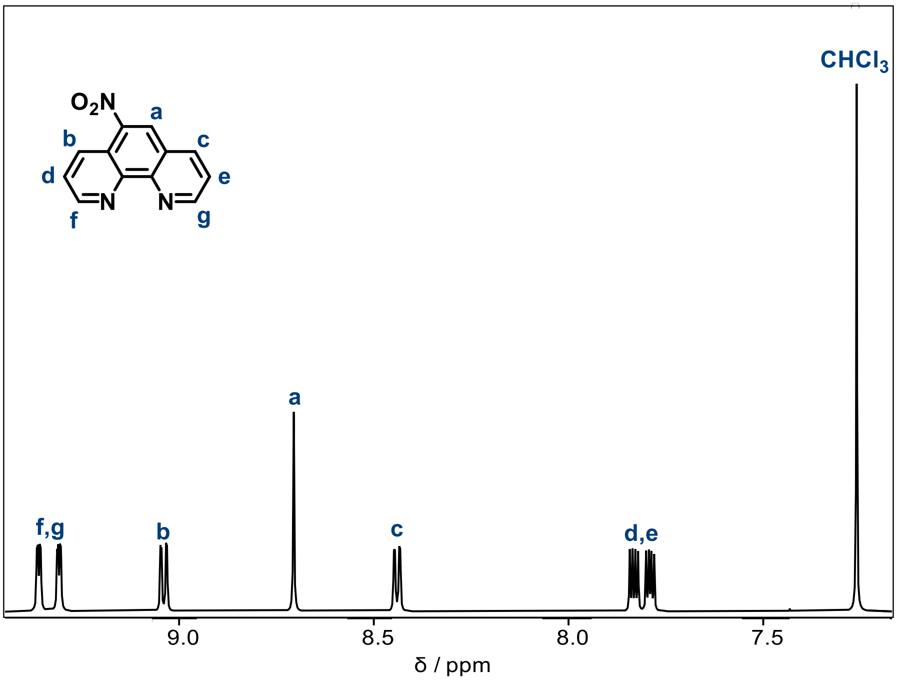

Figure S8. ^{1}H NMR (600 MHz, $CDCl_3$, 298 K) spectrum of **1**.

3.2 Synthesis of 5-amino-1,10-phenanthroline (2)

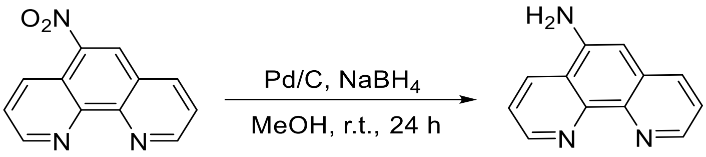

(2)

5-Amino-1,10-phenanthroline was synthesised as previously described with minor synthetic modifications.[3] 5-Nitro-2,2'-bipyridine (0.5 g, 2.48 mmol) was dissolved in methanol (100 mL) and the resulting solution was degassed by sparging with argon for 30 min. Subsequently, palladium on activated carbon (5 % Pd/C, 0.12 g) was added. The reaction mixture was cooled in an ice-bath, sodium borohydride (1.5 g) was added portion-wise under constant stirring. Following the complete addition of sodium borohydride, the ice bath was removed and the solution was stirred at room temperature for 5 h. The catalyst was filtered off with a celite-bed and the filtrate concentrated *in vacuo*. Water (100 mL) was added to the resulting mixture, followed

by an extraction with methylene chloride. The organic layer was dried with anhydrous $MgSO_4$. Subsequently, the filtrate was collected and concentrated under reduced pressure, affording a white solid. **Yield:** 0.38 g, 87%.

^{1}H NMR (600 MHz, DMSO-*d*$_6$, 298 K): δ (ppm) = 9.06 (dd, *J* = 4.2, 1.6 Hz, 1H), 8.71 – 8.65 (m, 2H), 8.05 (dd, *J* = 8.2, 1.7 Hz, 1H), 7.74 (dd, *J* = 8.3, 4.2 Hz, 1H), 7.51 (dd, *J* = 8.1, 4.2 Hz, 1H), 6.87 (s, 1H), 6.14 (s, 2H).**^{13}C NMR (150 MHz, DMSO-*d*$_6$, 298 K):** *δ* (ppm) = 102.2, 122.3, 122.5, 123.6, 131.0, 131.3, 133.2, 141.0, 143.2, 145.3, 146.7, 149.8. **LC-MS:** calculated: *m/z* = 196.0869 [M+H]$^+$; found: *m/z* = 196.0871 [M+H]$^+$.

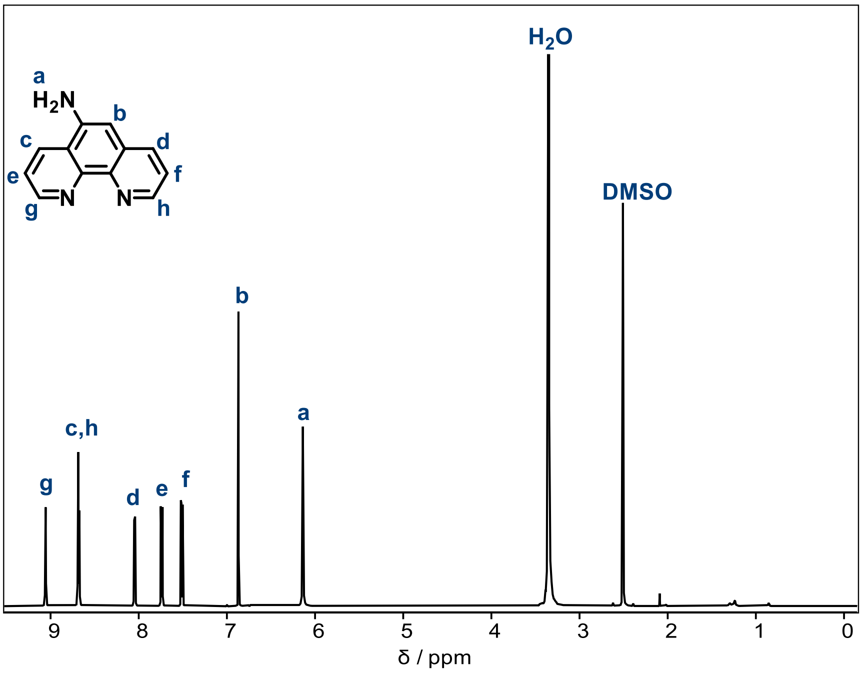

Figure S9. ^{1}H NMR (600 MHz, DMSO-*d*$_6$, 298 K) of **2** in DMSO-*d*$_6$.

3.3 Synthesis of *N*-(1,10-phenanthrolin-5-yl) methacrylamide (3)

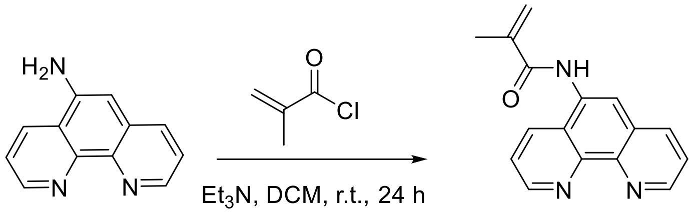

(3)

1,10-Phenanthrolin-5-amine (1.00 g, 5.12 mmol) was dissolved in 30 mL of dry dichloromethane and cooled to 0 °C under nitrogen. Triethylamine (2.36 mL, 16.9 mmol) was added, and the mixture was stirred for 30 min. Subsequently, methacrylic chloride (0.85 mL, 8.71 mmol) was slowly added and a gas formation was observed. The solution was stirred for 2 h at 0 °C. Next, the solution was warmed up to room temperature. Subsequently, the reaction mixture was stirred overnight. The resulting solution was filtered and then washed sequentially with 5% aqueous sodium bicarbonate and water. The resulting solid was dried in vacuum and a pale-yellow crystal solid was obtained. **Yield:** 1.10 g, 75%.

^{1}H NMR (600 MHz, DMSO-d_6, 298 K): δ (ppm) = 10.17 (s, 1H), 9.13 (m, 2H), 8.49 (m, 2H), 8.04 (s, 1H), 7.81 (o, 2H), 6.08 (s, 1H), 5.65 (s, 1H), 2.07 (s, 3H). **^{13}C NMR (150 MHz, DMSO-d_6, 298 K):** δ (ppm) = 19.3, 121.3, 123.0, 123.4, 124.0, 126.2, 128.4, 132.5, 132.7, 136.4, 140.2, 144.7, 146.3, 150.1, 150.4, 168.3. **LC-MS:** calculated: *m/z* = 264.1137 [M+H]$^+$; found: *m/z* = 264.1133 [M+H]$^+$.

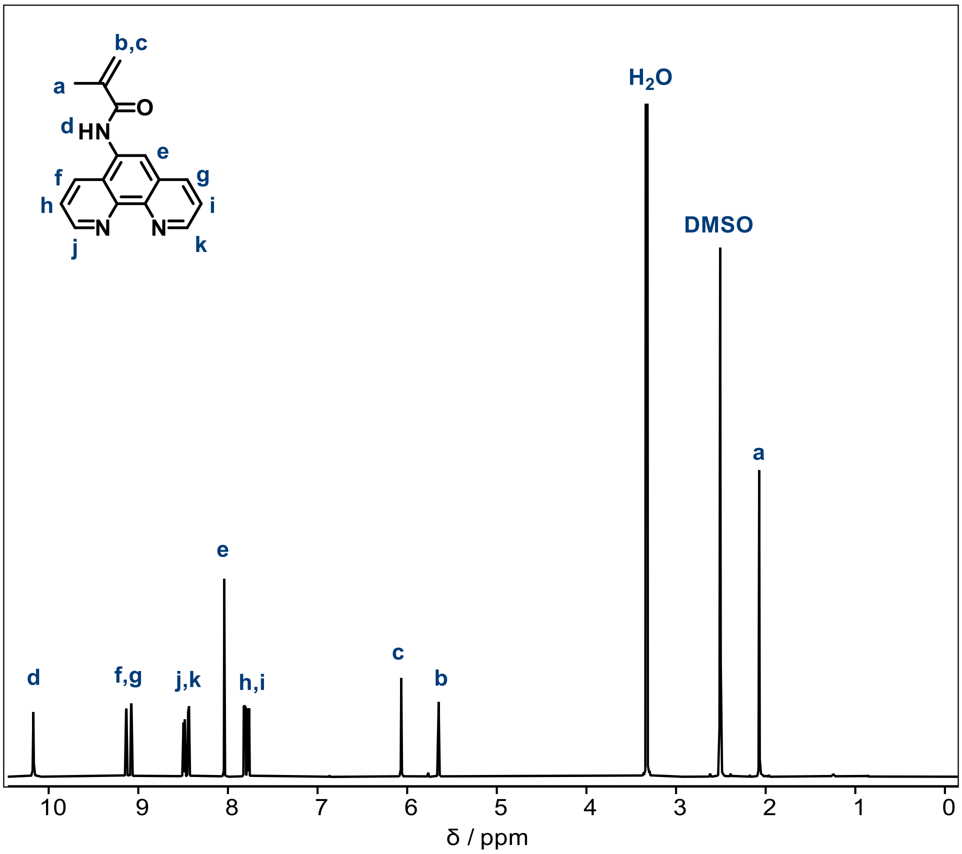

Figure S10. ^{1}H NMR spectrum (600 MHz, DMSO-d_6, 298 K) of **3**.

3.4 Synthesis of bis(2,2′-bipyridine)-(1,10-phenanthrolin-5-yl-methacrylamide)ruthenium(II) bis(hexafluorophosphate) (Ru(II)-CM) (4)

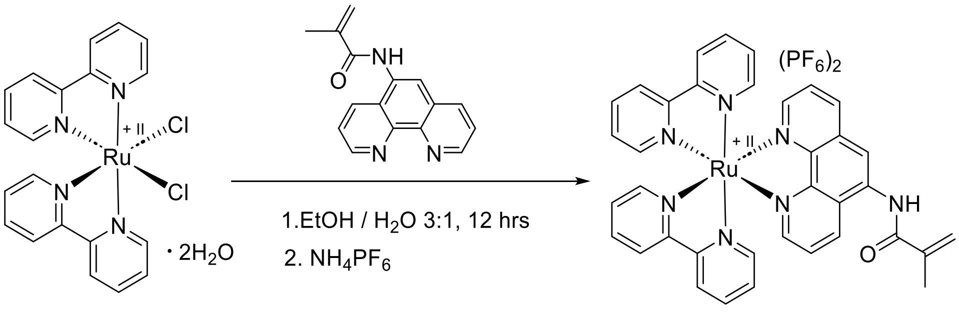

(4)

Bis(2,2′-bipyridine)(1,10-phenanthrolin-5-yl)acrylamide ruthenium(II) hexafluoro-phosphate was prepared using a modified literature procedure.[4] Herein, dichloridobis(2,2'-bipyridine)ruthenium(II) (66 mg, 0.170 mmol) was dissolved in 5 mL of EtOH/H_2O (ratio 3:1 v/v). Then, *N*-(1,10-phenanthrolin-5-yl) methacrylamide (50 mg, 0.170 mmol) was added to the solution. Subsequently, the reaction mixture was

refluxed overnight and a color change from purple to orange occurred. After the reaction was cooled to ambient temperature, ammonium hexafluorophosphate (56 mg, 0.340 mmol) was added to the mixture. The resulting solid was washed with water (2 x 10 mL) and diethyl ether (2 x 10 mL). The solid was dried under reduced pressure. An orange powder was obtained. **Yield:** 0.065g, 78%.

^{1}H NMR (600 MHz, DMSO-*d*6 , 298 K): δ (ppm) = 10.44 (s, 1H), 8.90 – 8.82 (m, 4H), 8.75 (ddd, J = 21.7, 8.4, 1.2 Hz, 2H), 8.49 (s, 1H), 8.24 – 8.06 (m, 6H), 7.91 – 7.83 (m, 4H), 7.59 (ttd, J = 6.4, 3.1, 1.2 Hz, 4H), 7.37 (tdd, J = 7.7, 5.8, 1.3 Hz, 2H), 6.09 – 6.06 (m, 1H), 5.72 (d, J = 1.7 Hz, 1H), 2.10 – 2.05 (m, 3H). **^{13}C NMR (150 MHz, DMSO-*d*6, 298 K):** δ (ppm) = 18.67, 121.59, 122.05, 124.42, 125.82, 126.56, 127.32, 127.84, 129.98, 133.53, 133.96, 136.42, 137.86, 139.51, 145.01, 147.12, 151.37, 151.51, 152.42, 156.59, 156.81, 167.96. **LC-MS:** calculated: m/z = 676.1477 $[M+H]^+$; found: m/z = 676.1430 $[M+H]^+$.

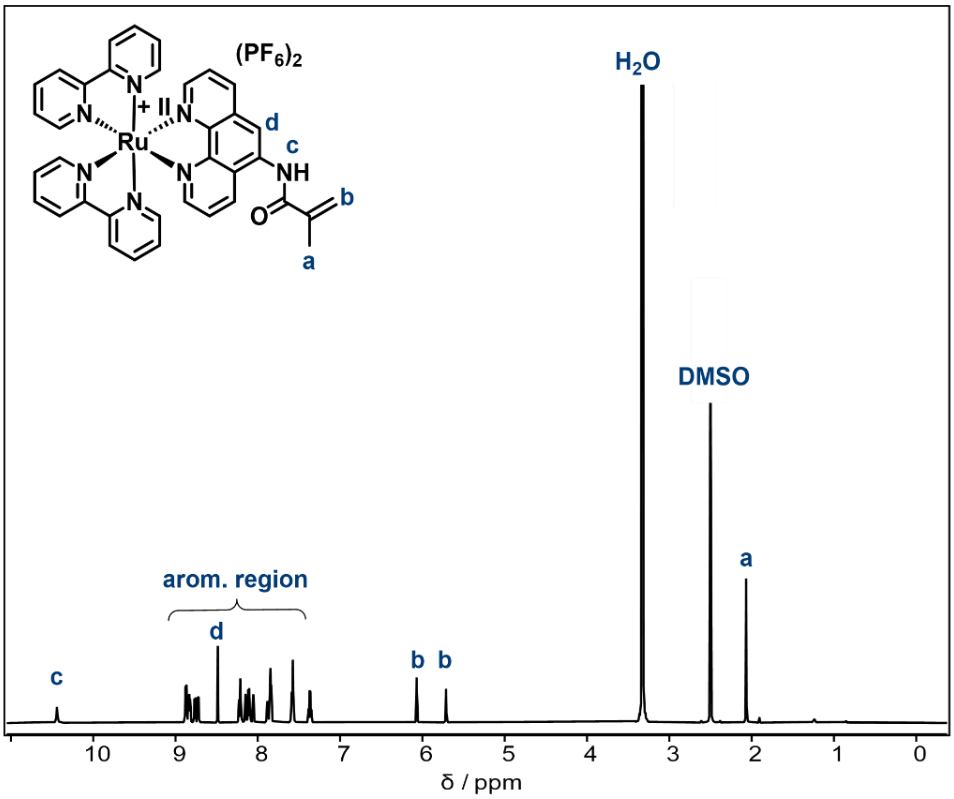

Figure S11. ^{1}H NMR spectrum (600 MHz, DMSO-*d*6, 298 K) of **4**.

4.1 Irradiation set-up used for catalytic experiments

For all irradiation experiments the following setup was used:

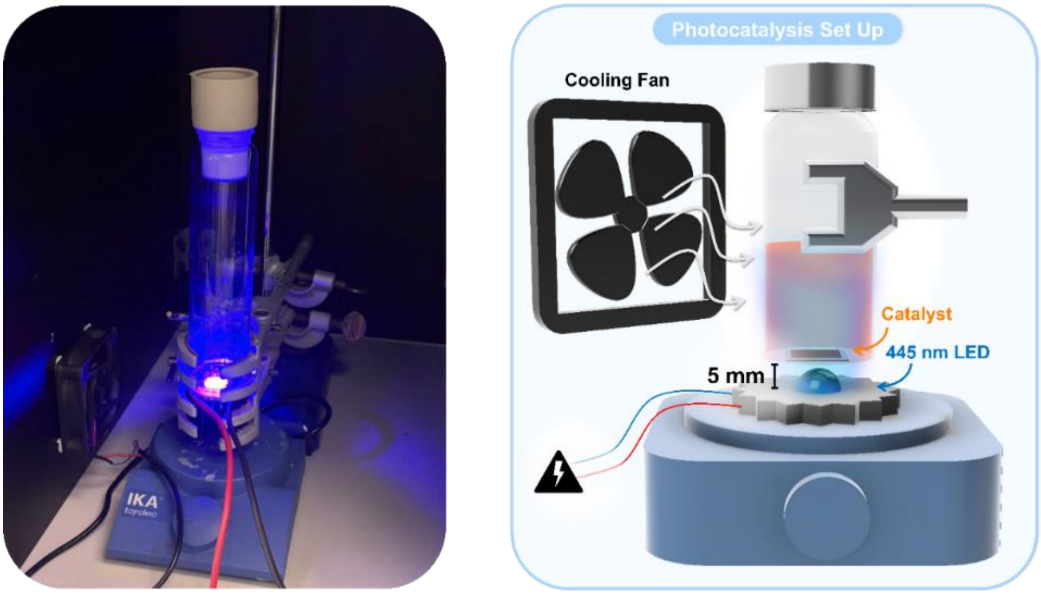

Figure S12. Irradiation setup for photoreactions. The maximum emission wavelength of the LED is 445 nm.

4.2 Printed structures for catalytic experiments

To test the catalytic reactivity, mesh structures were printed using both SLA and DLW. Supplementary File 5 provides the G-code file for the SLA printed mesh.

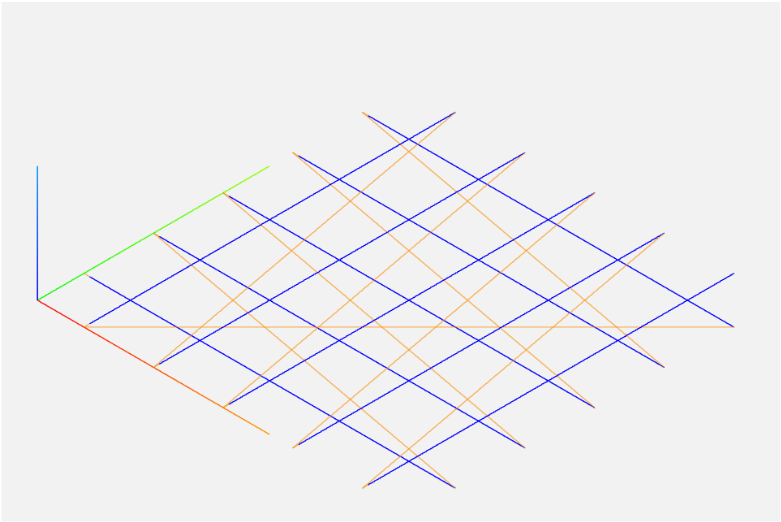

Figure S13. (X, Y, and Z) stage trajectories for the mesh structure, generated from the G-code files provided in Supplementary Files 2-4. Blue lines represent the trajectory during laser exposure (shutter open), while orange lines indicate movement with the laser shutter closed. The printed structure can be seen in Figure S15.

4.3 Molecular Catalysis

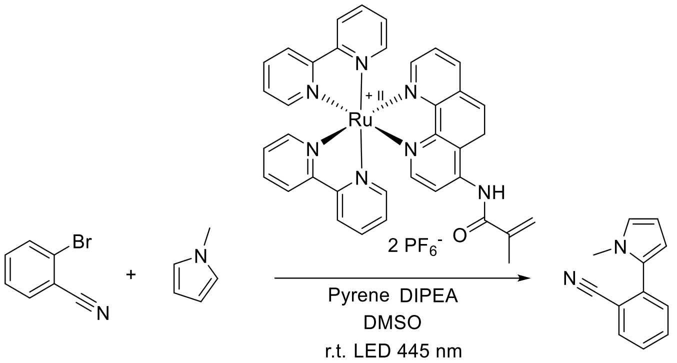

The photocatalytic reaction was carried out as previously described with minor synthetic modifications.[5] A solution was prepared by dissolving pyrene (0.2 mg, 1 μmol), bis(2,2′-bipyridine)(1,10-phenanthrolin-5-yl)acrylamide ruthenium(II) hexafluorophosphate (0.97 mg, 1 μmol), and 2-bromobenzonitrile (5 mg, 80 μmol) in 0.9 mL of DMSO-*d*6. The resulting mixture was placed in a crimped 10 mL septum vial and sparged with argon for 10 min. Subsequently, a solution of *N*-methyl pyrrole (65 mg, 800 μmol) and DIPEA (13 mg, 100 μmol) in 0.1 mL of DMSO-*d*6 was added to the reaction mixture and the resulting solution was then sparged with argon for 5 min. The vial was placed 5 mm above an LED (445 nm, 10 W, S1) and irradiated for 60 min (refer to Figure S11). Reaction conversion was calculated from the integral values of the proton resonances at 6.16, 6.34, 6.97, based on the resonance at 7.94 ppm in the ^{1}H NMR spectra (Section 4.3). A control experiment, conducted in the absence of the Ru(II) catalyst, resulted in no conversion (Figure S14).

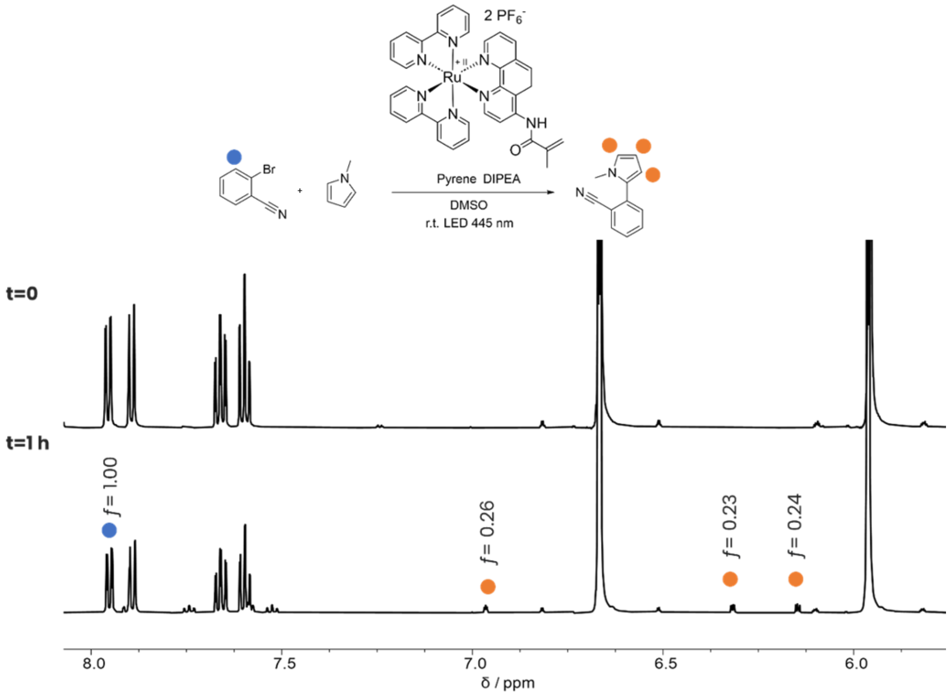

Figure S14. Expanded ^{1}H NMR (600 MHz, DMSO-*d*6, 298 K) spectra of the reaction mixture at *t*=0, and crude reaction solution after 1 h of irradiation time. The blue and orange marks represent proton resonances assigned to 2-bromobenzonitrile (starting

material) and 2-(1-methyl-1*H*-pyrrol-2-yl)benzonitrile (product), with the corresponding integrals of the assigned resonances. Herein **Ru(II)-CM** was used as a catalyst.

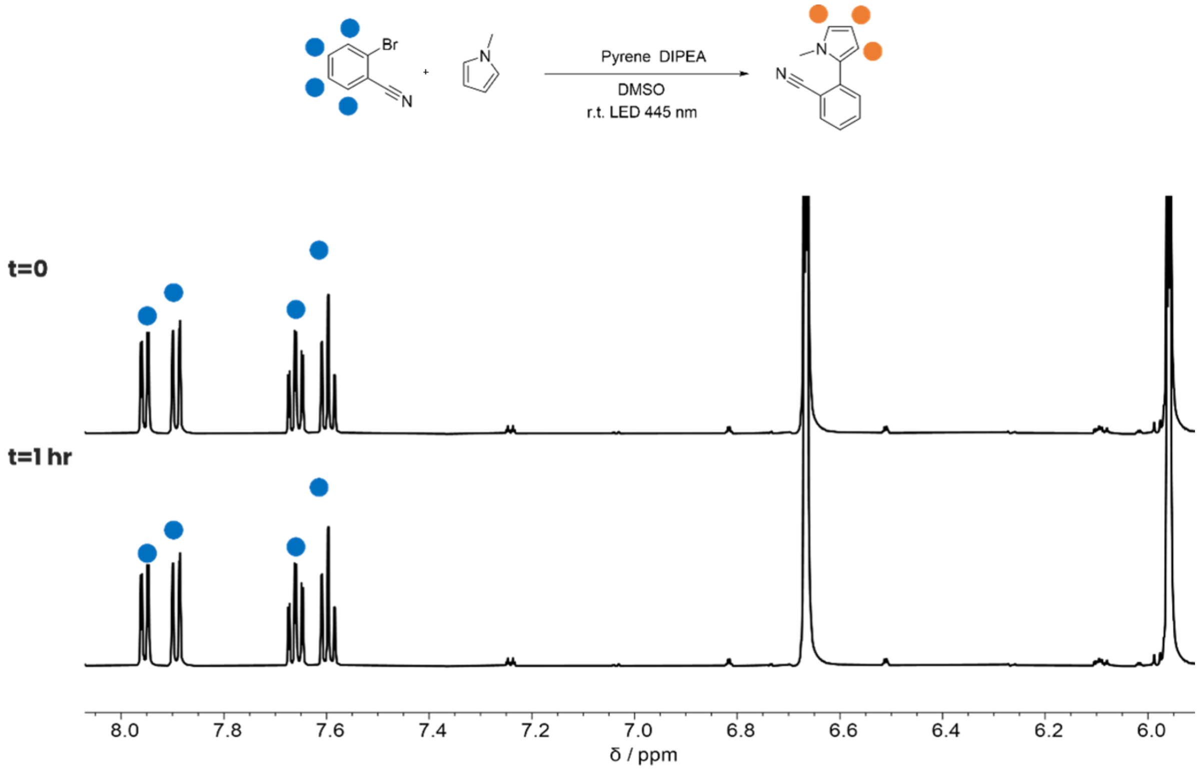

Figure S15. Control experiment without the use of a Ru(II) catalyst. Expanded ^{1}H NMR(600 MHz, DMSO-d_6, 298 K) spectra of the reaction mixture at *t*=0, and crude reaction solution after 1 h of irradiation time. No product was formed and only the starting material (2-bromobenzonitrile) is marked in blue.

4.4 Catalysis with 3D Printed Mesh Structures

Both the SLA-printed and DLW-printed structures were evaluated for catalytic activity using the procedure described in Section 4.3. A larger crimp vial was required to fit the catalysts. 2-Bromobenzonitrile (80 µmol, 15 mg) dissolved in 0.9 mL of DMSO-d_6 was added. The resulting mixture was sparged with argon for 10 min. Thereafter, *N*-methyl pyrrole (800 µmol, 65 mg) and DIPEA (100 µmol, 13 mg) were dissolved in 0.1 mL of DMSO-d_6 and the mixture was added into the solution. After purging the solution with argon for 5 min, the vial was placed 5 mm above an LED (455 nm, 10 W, refer to Figure S1 for emission spectrum) and irradiated for 60 min. Figure S15 (a-b) shows the SLA-printed catalysts before and after the experiment. For the DLW-printed structure an SEM picture (S16, S17) is only available after the experiment, as the platinum coating required for the SEM measurement would cover the surface of the catalyst, however, even after the experiment the structure is well maintained.

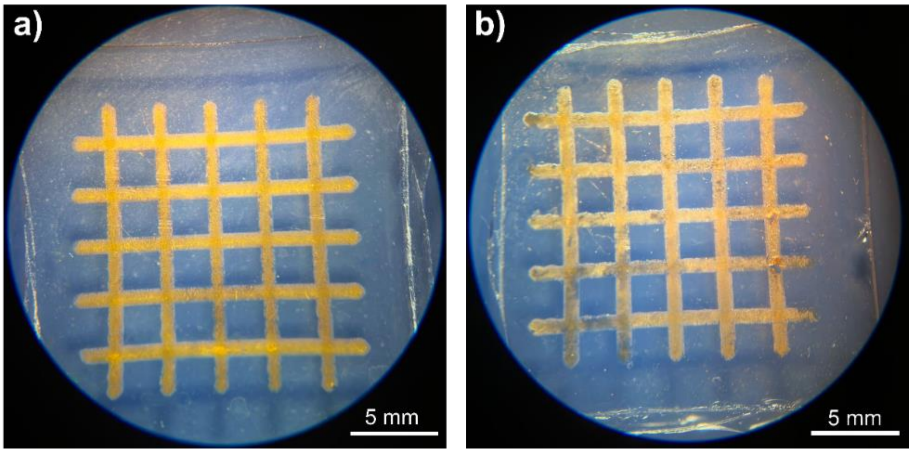

Figure S16. Photographs of the SLA printed mesh structure before (a) the usage in the photocatalytic reaction and (b) after the reaction.

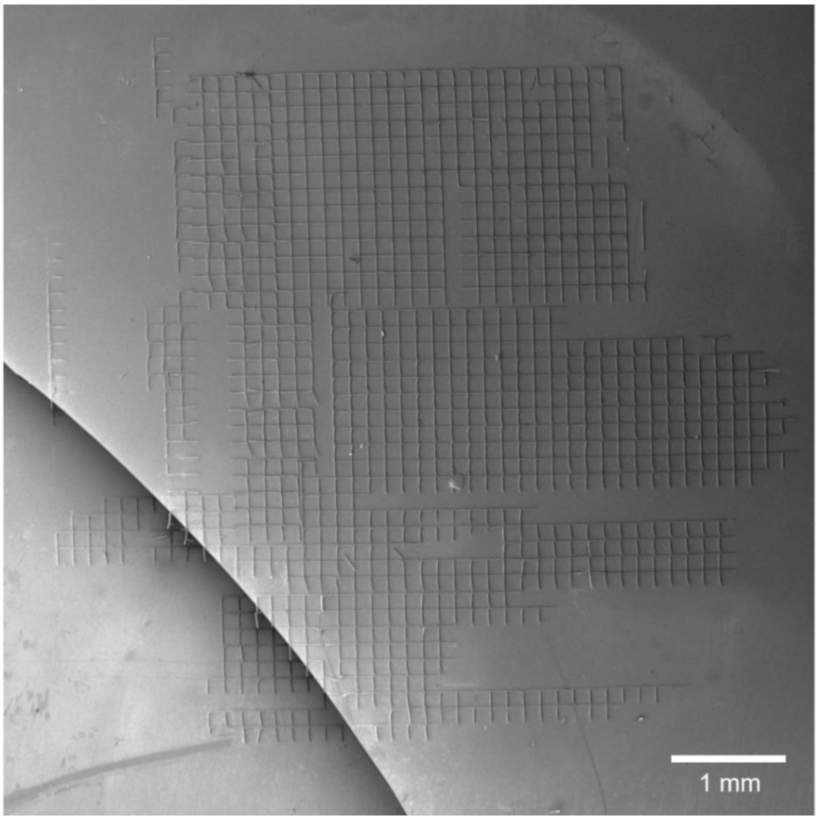

Figure S17. SEM image of the DLW printed catalyst used in catalytic reaction. The dark line in the picture stems from a glass brakeage of the substrate where the structure is printed on, which occurred during the coating of the sample for SEM after the usage as a catalyst. The uneven print of the mesh stems from the small printing window and is due to printing on several different days making a perfect stitching of the printing units not possible. The entire print took an approximate 8 h to print.

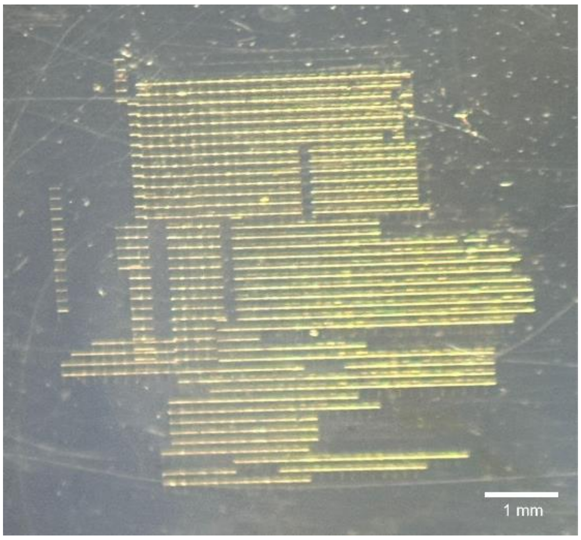

Figure 18. Microscopic image of the DLW-printed catalyst before the usage as a catalyst.

4.5 Calculation of Conversion

Reaction conversion of the photoredox reaction was calculated using integrals of the peaks assigned to the starting material (7.94 ppm) and products (6.16, 6.34, 6.97 ppm). The solvent signal was used as internal standard. An example of the calculation is below:

Conversion / % = (Ave. 0.47+0.45+0.47) / [1.00 + (Ave. 0.47+0.45+0.47)]

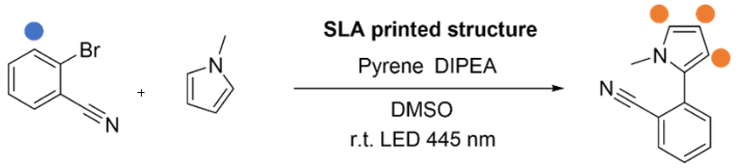

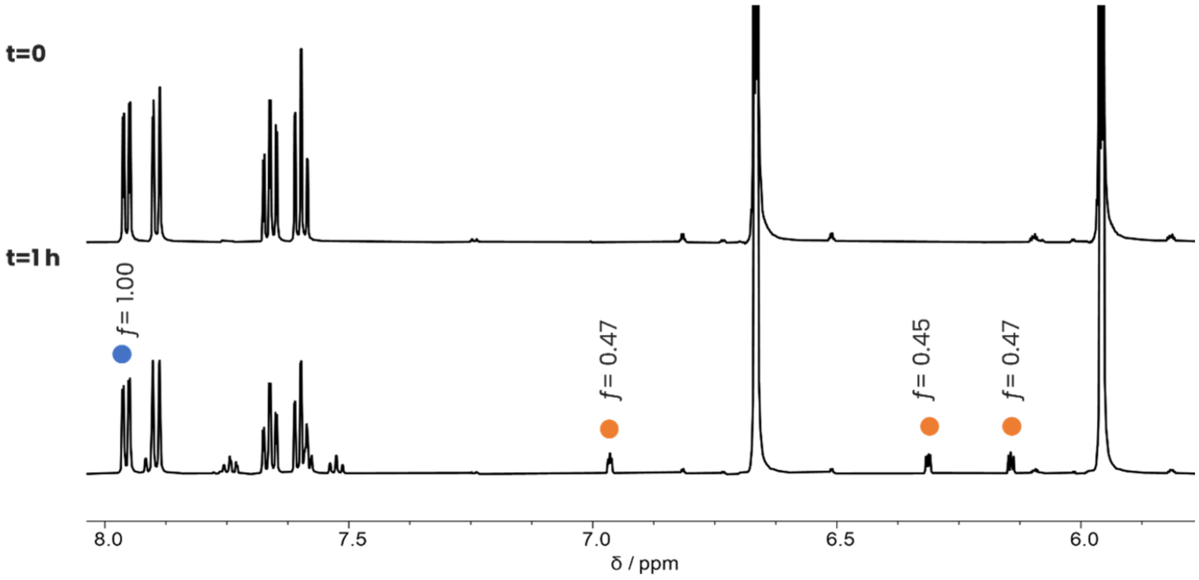

Figure S19. Expanded ^{1}H NMR (600 MHz, DMSO-d_6, 298 K) spectra of the reaction mixture at *t*=0, and crude reaction solution after 1 h of irradiation time. The blue and orange marks represent proton resonances assigned to 2-bromobenzonitrile (starting

material) and 2-(1-methyl-1*H*-pyrrol-2-yl)benzonitrile (product), with the corresponding integrals of the assigned resonances.

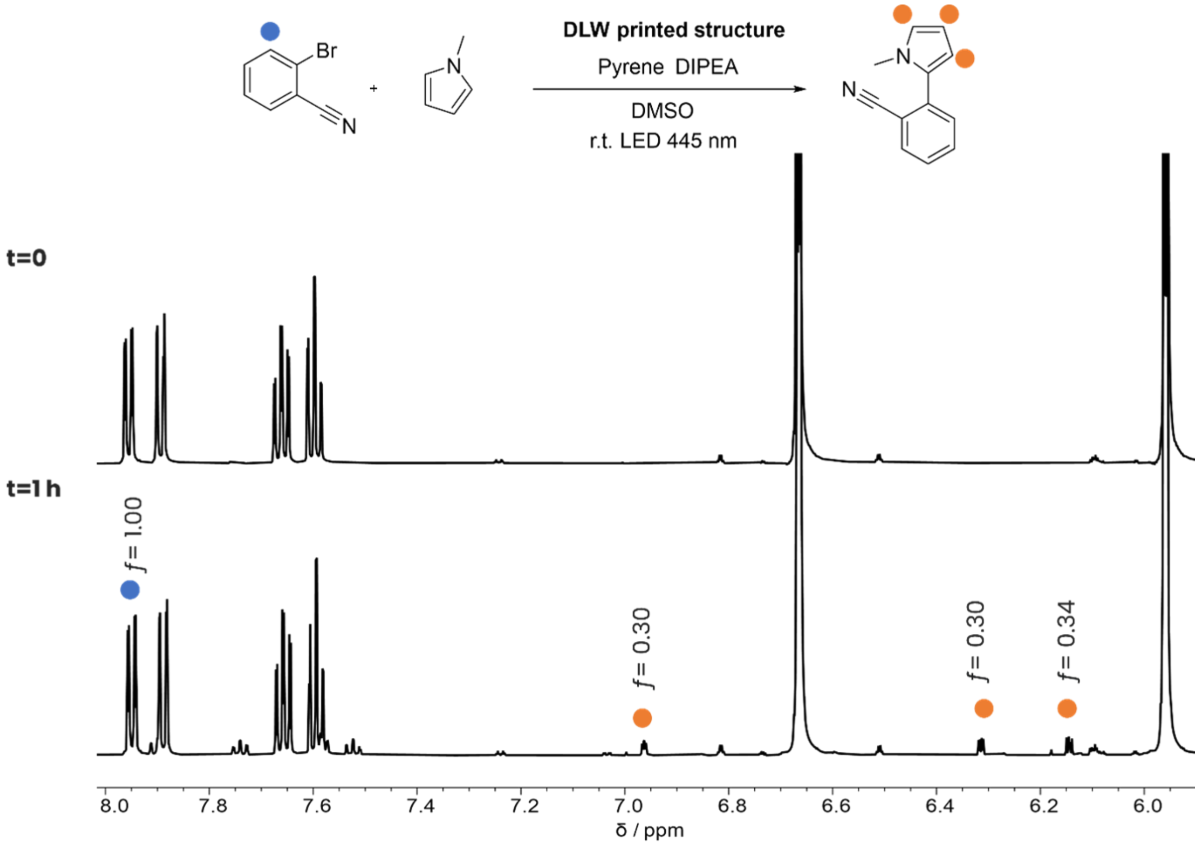

Figure S20. Expanded ^{1}H NMR (600 MHz, DMSO-d_6, 298 K) spectra of the reaction mixture at *t*=0, and crude reaction solution after 1 h of irradiation time. Herein, the DLW print was used.

4.6 Recycling Catalysis Studies with DLW Printed Mesh

Table S6. To demonstrate the printed catalyst's recyclability, the DLW-printed photocatalyst was used in five photocatalysis cycles with a DMSO rinse in between. All reaction conditions are identical. Note that after the 4th cycle detachment of some of the printed lines was visible after the DMSO washing step.

**Photocatalysis Cycle**	***Conversion***
1	27.7%
2	24.1%
3	23.8%
4	22.8%
(5	18.7%)

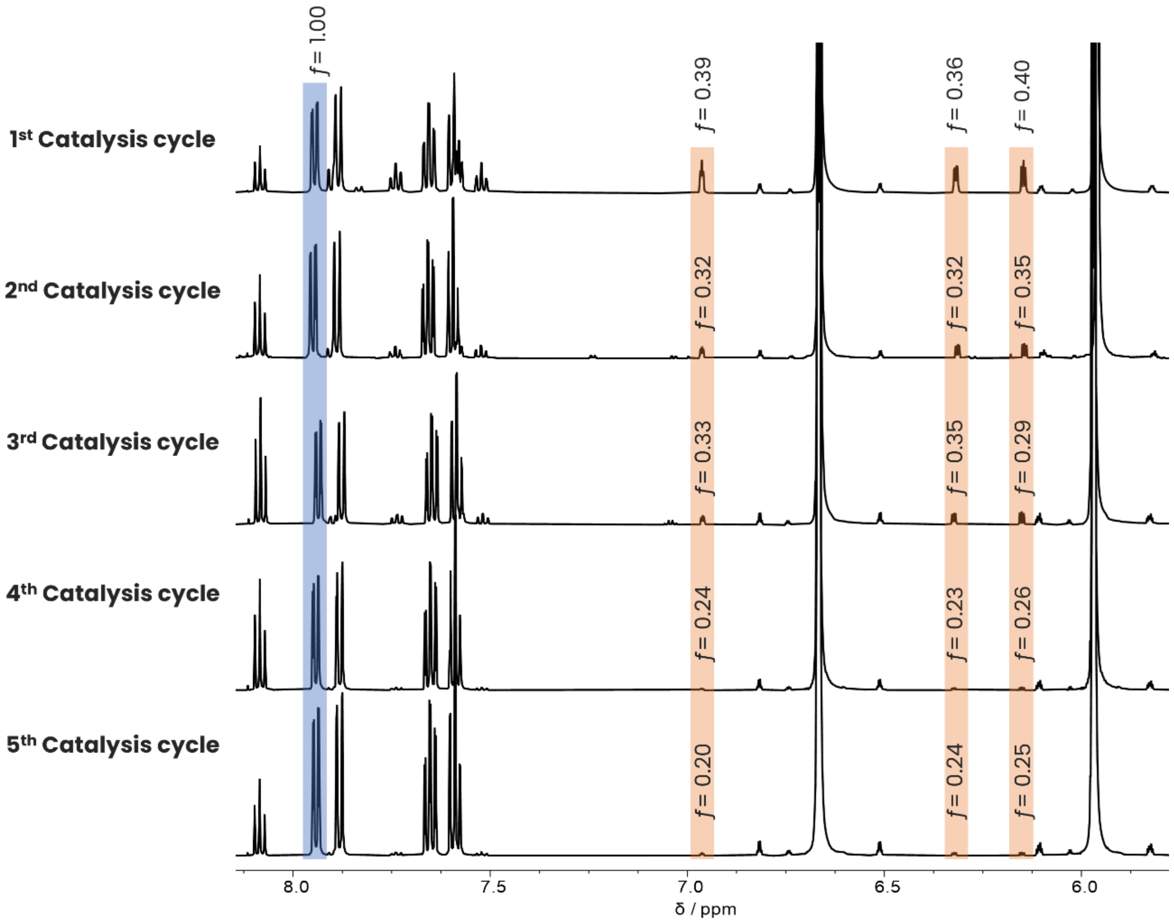

Figure S21. Expanded ^{1}H NMR (600 MHz, DMSO-d_6, 298 K) spectra of the reaction mixtures after each photocatalysis cycle. The blue and orange marks represent proton resonances assigned to 2-bromobenzonitrile (starting material) and 2-(1-methyl-1*H*-pyrrol-2-yl)benzonitrile (product), with the corresponding integrals of the assigned resonances.

4.7 Calculation of the Surface of both the SLA and DLW Mesh

SLA Print

The surface calculation of the structure is only approximate, as the surface is rough and inconsistent as seen in Figure S21. To determine the height, we imaged the structure from the side to obtain a profile. Subsequently, 15 points were selected and an average height was determined.

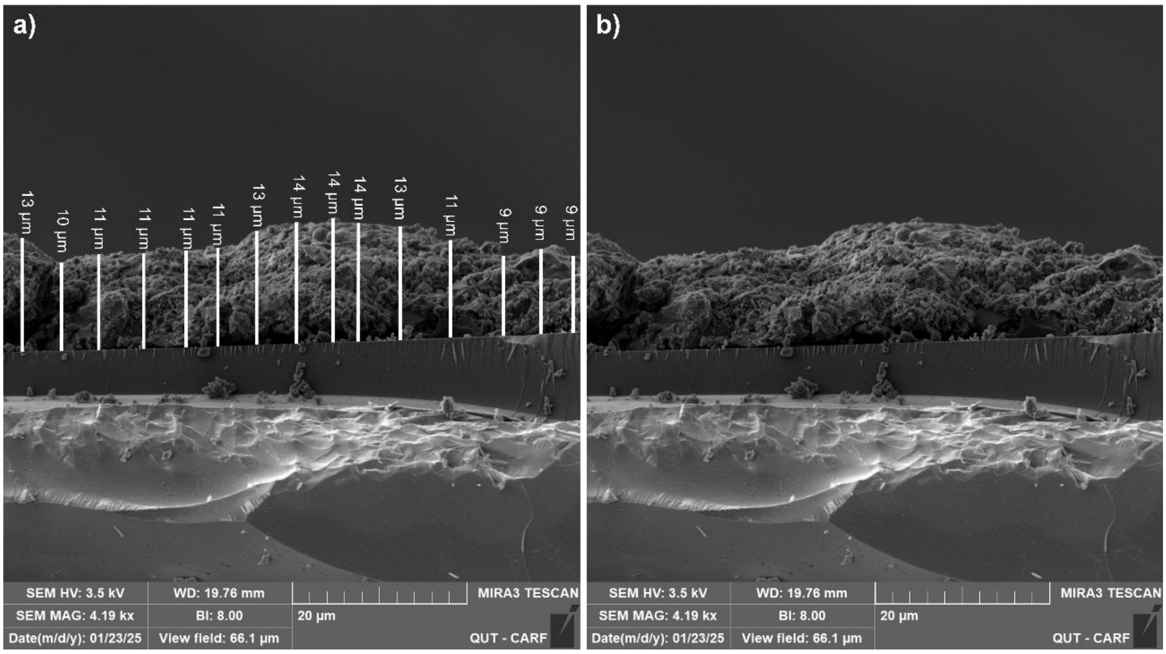

Figure S22. SEM picture of the side profile of the SLA mesh. (a) Elucidates how the height was calculated (b) shows the same structure without the overlay.

Next, using the average height and the length and thickness of each line, the surface of a line was calculated as per Figure S22. The structure was simplified as the edges in the actual print are rounded. Further, the crossing sections of the 10 lines are disregarded. The exact numbers of the height calculation can be found in Table S5. A single line measures 12.29 mm^2. Multiplication by 10 (number of lines refer to Figure S14) affords a total of 123 mm^2 for the entire surface of the printed mesh. The volume of the entire print measures 1.239 mm³ (all sides multiplied, this again multiplied by the number of lines).

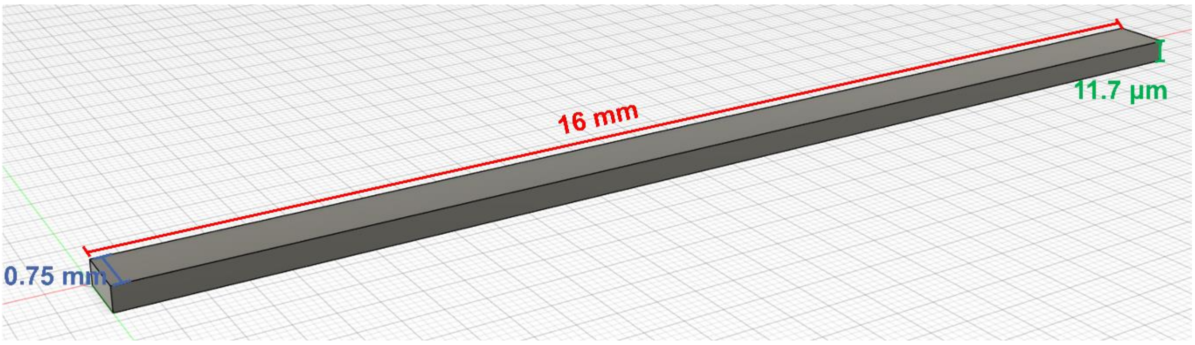

Figure S23. Measurements that were used to calculate the surface of the SLA printed mesh.

Table S7. Exact values that were used to calculate the average height of the SLA-printed structure.

Measurement Spot	Height [μm]
1	13.25
2	10.17
3	11.09
4	11.09
5	11.15
6	11.39
7	13.18
8	14.03
9	14.41
10	13.71
11	13.20
12	11.28
13	9.29
14	9.83
15	8.92

DLW Print

For the DLW-mesh, we counted each printed unit, with a printing unit consisting of the structure depicted in Figure S23. The entire structure can be seen in Figure S16. The size of the units was taken from the STL file which was used for printing, refer to Figure 23 for all the measurements. Each unit has a surface of 7475 μm^2 after multiplying with 1037 and conversion to mm^2, we calculated a surface area of 7.8 mm^2. The volume of the print overall measures 0.0153 mm^3.

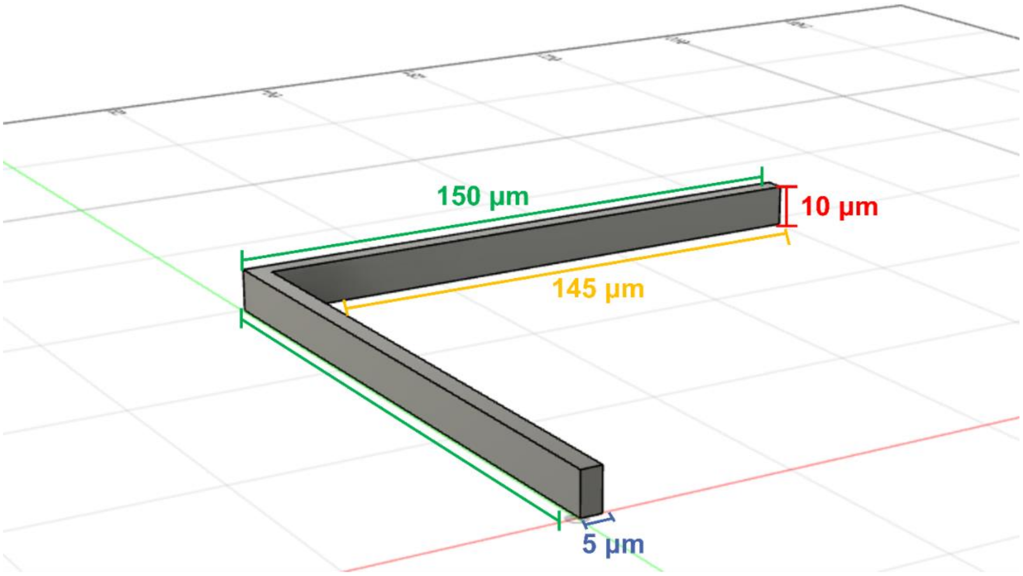

Figure S24. Dimensions of the single unit that was printed to build the DLW-mesh. This design was chosen as it can be optimized for rapid printing conditions. Arrays were printed with a scanning speed of 125000 μm s^{-1} and the entire structure took approximately 8 h to print.

5 Analytical Data

5.1 Nuclear Magnetic Resonance (NMR) Spectra

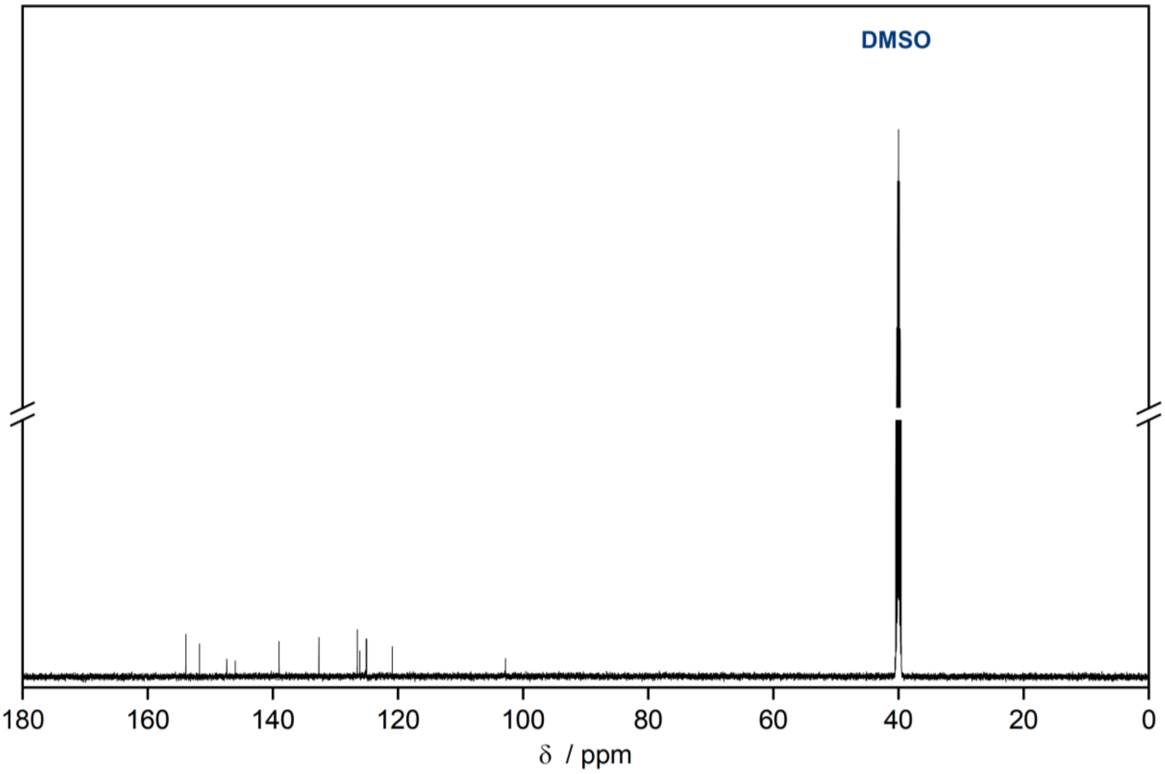

Figure S25. ^{13}C NMR (150 MHz, DMSO-d_6, 298 K) spectrum of **1**.

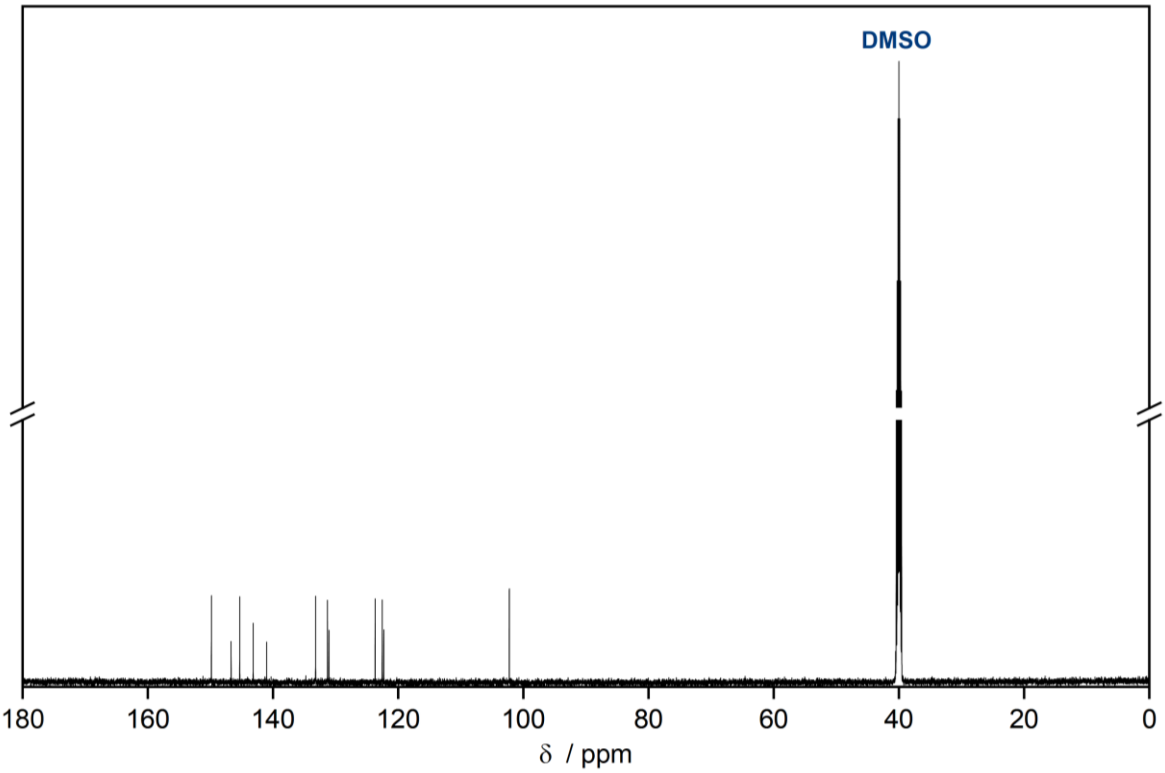

Figure S26. ^{13}C NMR (150 MHz, DMSO-d_6, 298 K) spectrum of **2**.

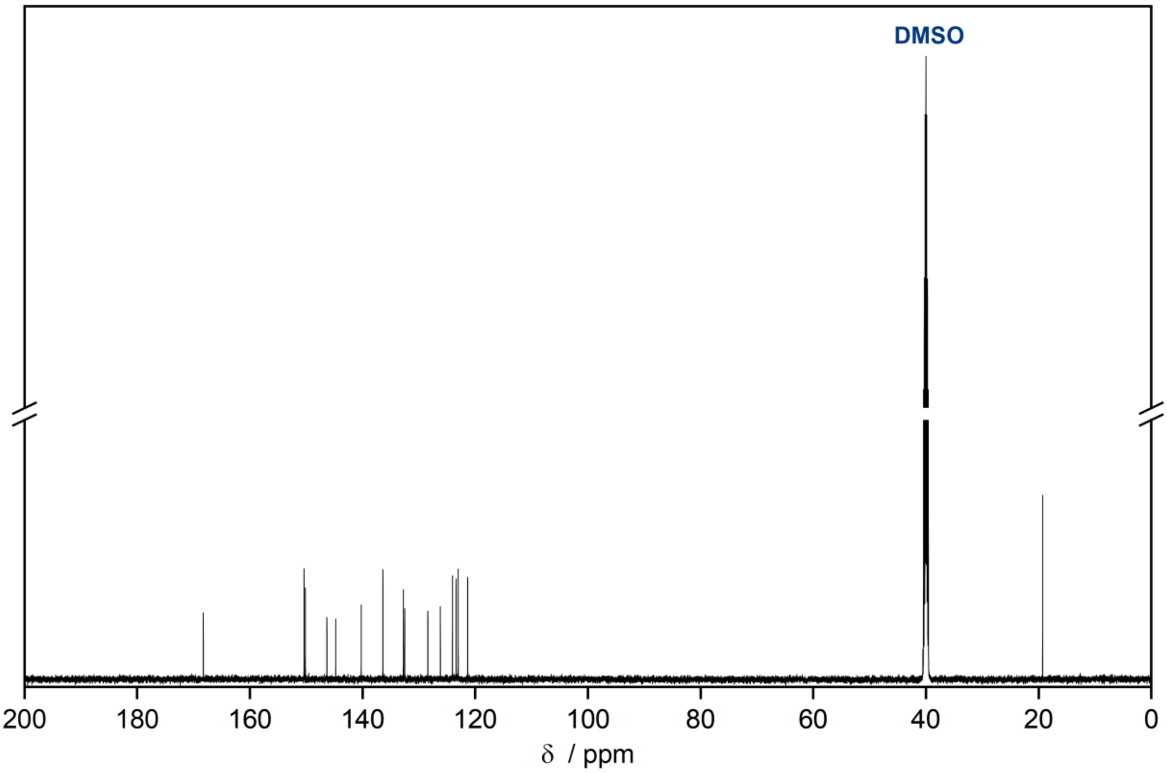

Figure S27. ^{13}C NMR (150 MHz, DMSO-d_6, 298 K) spectrum of **3**.

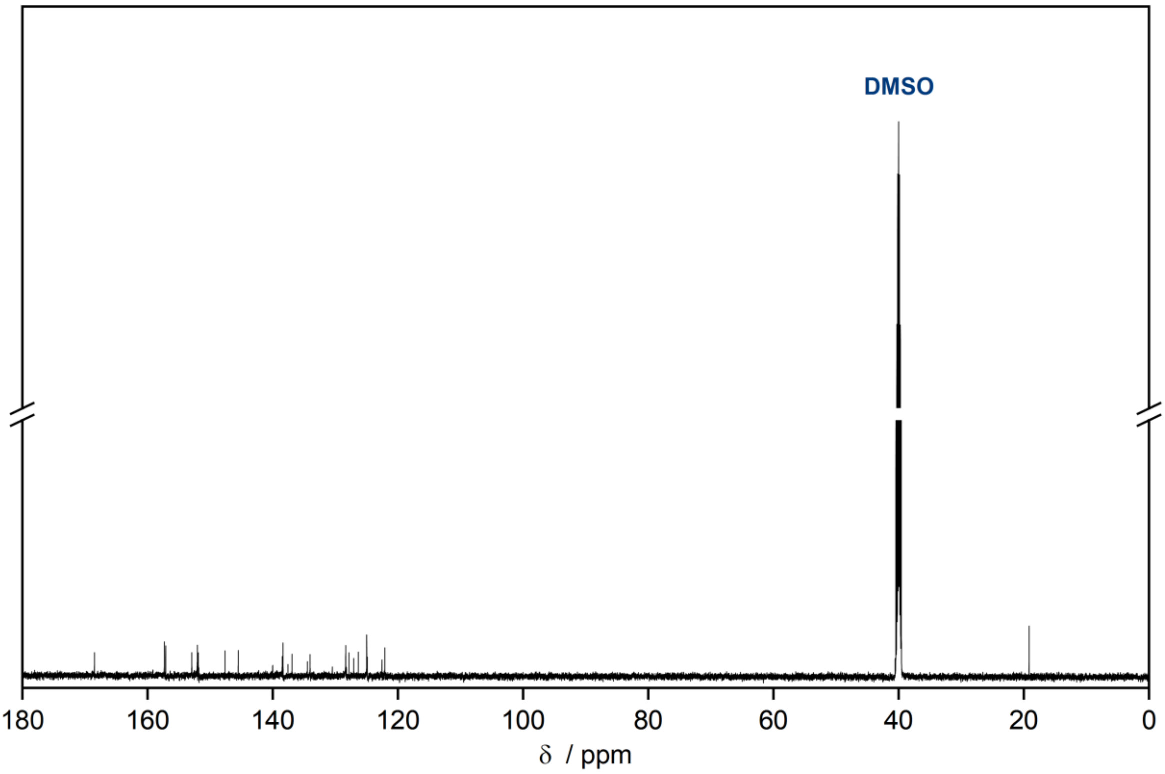

Figure S28. ^{13}C NMR (150 MHz, DMSO-d_6, 298 K) spectrum of **4**.

5.2 Mass Spectrometry

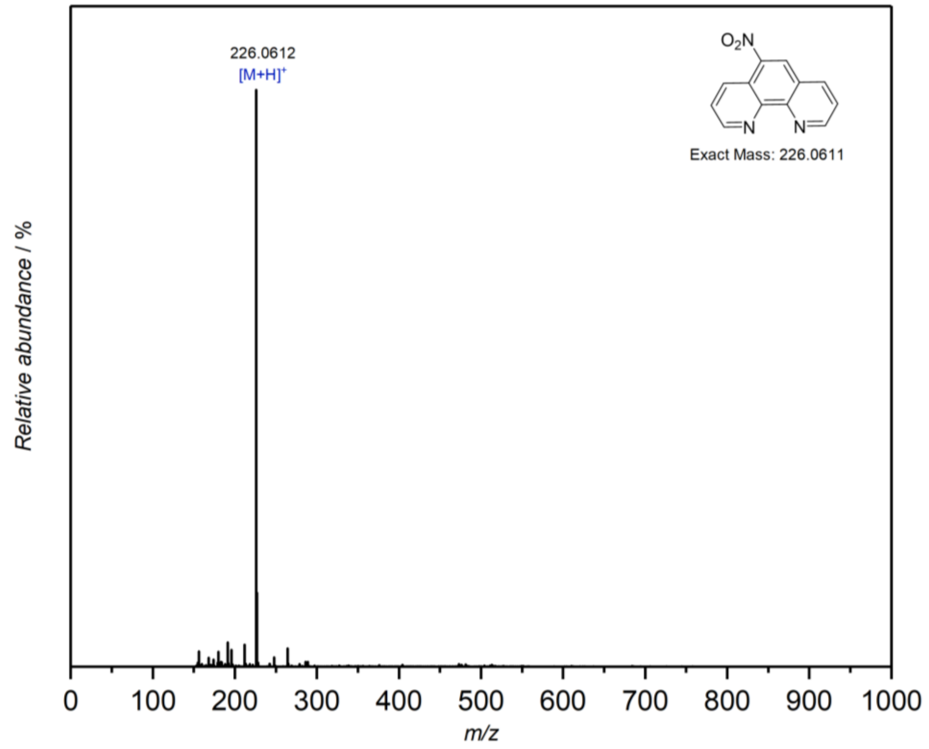

Figure S29. ESI mass spectrum (0.01 mg mL^{-1} in acetonitrile) of **1**.

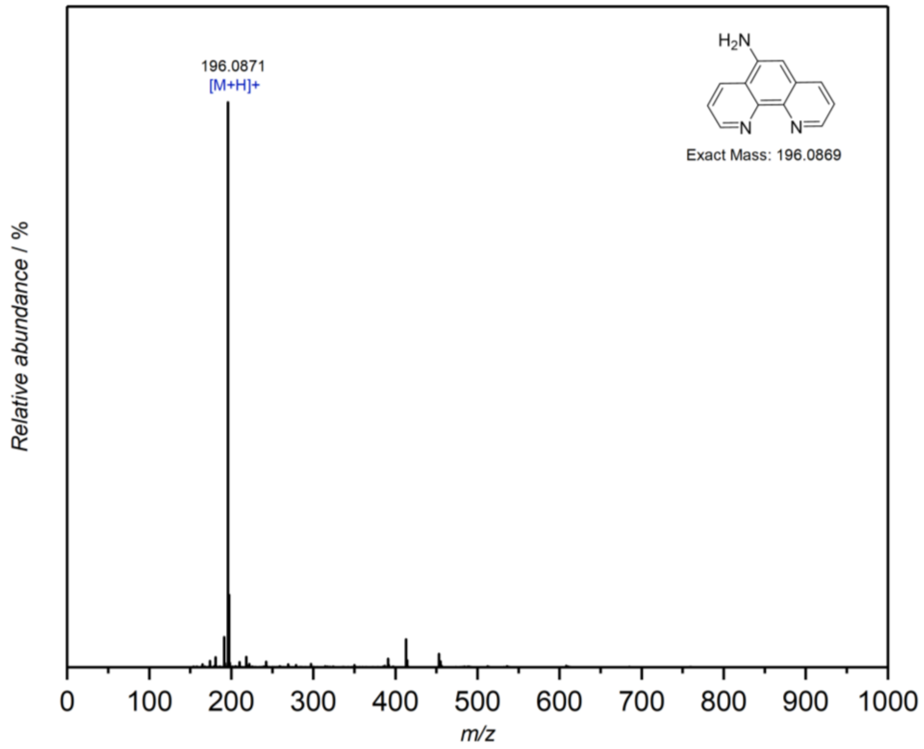

Figure S30. ESI mass spectrum (0.01 mg mL^{-1} in acetonitrile) of **2**.

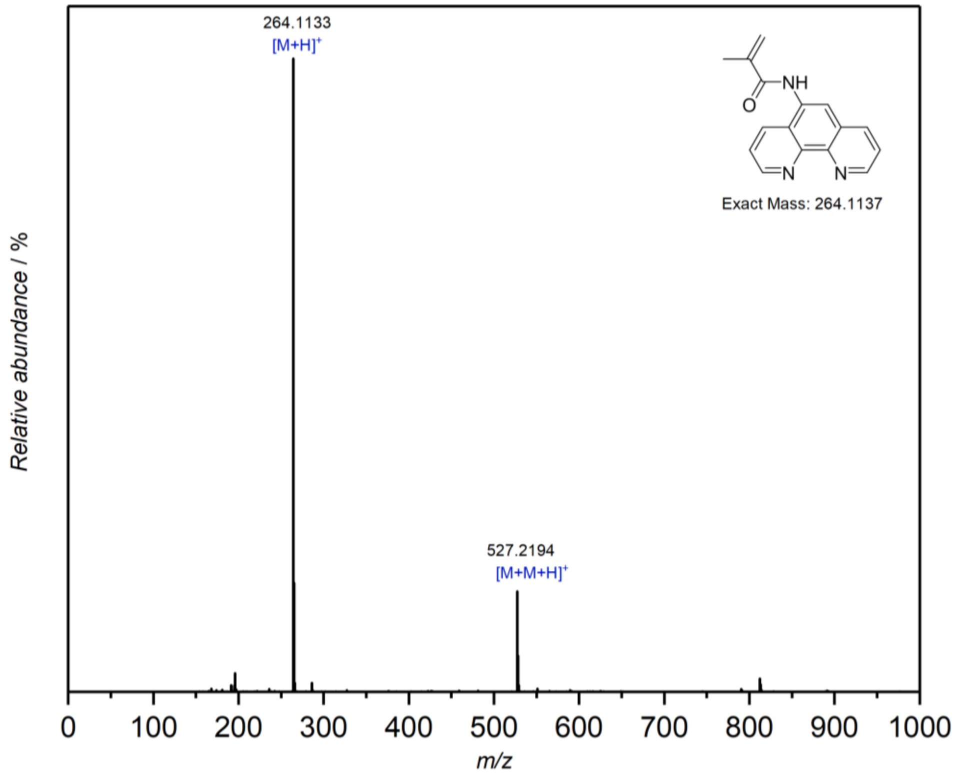

Figure S31. ESI mass spectrum (0.01 mg mL^{-1} in acetonitrile) of **3**.

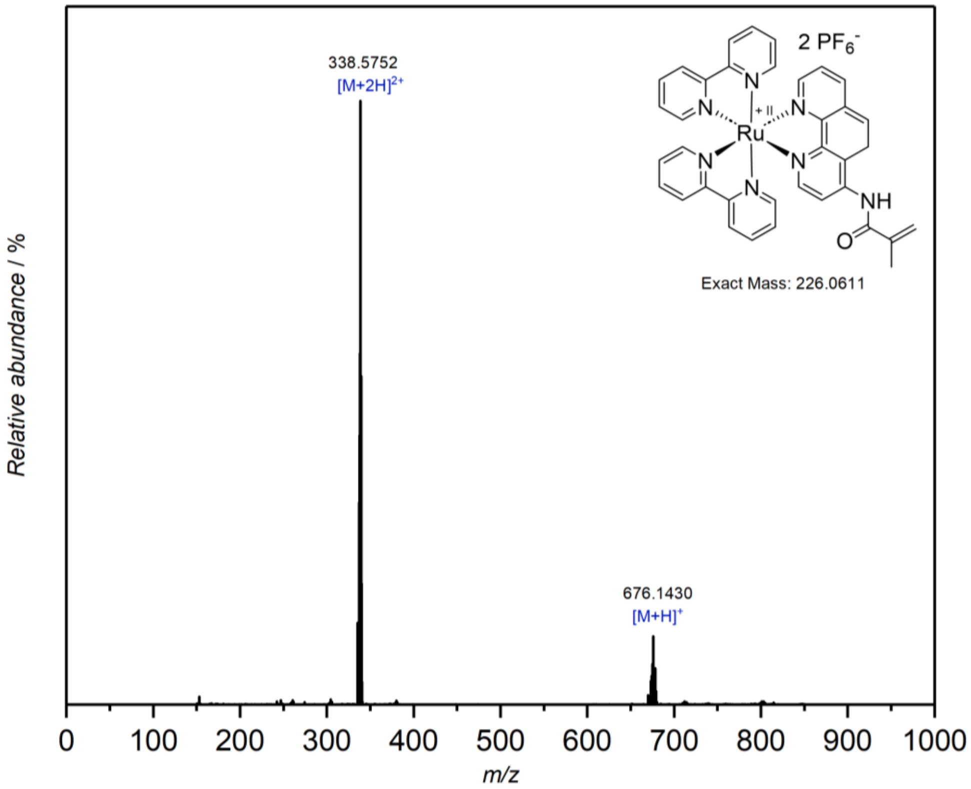

Figure S32. ESI mass spectrum (0.01 mg mL^{-1} in acetonitrile) of **4**.

5.3 Additional ToF-SIMS Images

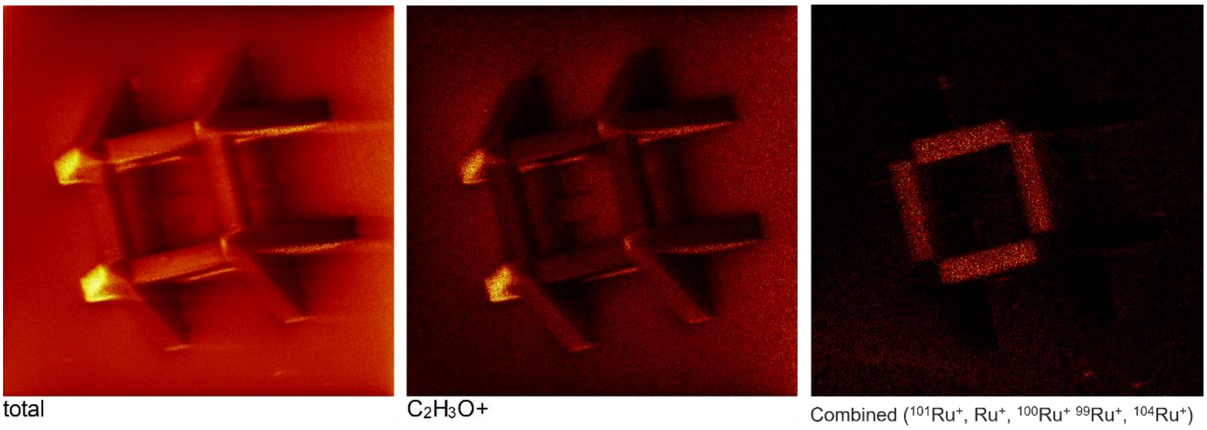

Figure S33. Additional ToF-SIMS images of the multi-material boxing-ring structure. The corresponding ions are depicted underneath each structure.

References

1. P. Kiefer, V. Hahn, M. Nardi, L. Yang, E. Blasco, C. Barner-Kowollik and M. Wegener, *Advanced Optical Materials*, **2020**, 8, 2000895.
2. X. Wu, K. Ehrmann, C. T. Gan, B. Leuschel, F. Pashley-Johnson and C. Barner-Kowollik, *Adv. Mater.*, **2025**, *37*, 2419639
3. S. Ji, H. Guo, X. Yuan, X. Li, H. Ding, P. Gao, C. Zhao, W. Wu, W. Wu and J. Zhao, *Org. Lett.*, **2010**, 12, 2876-2879.
4. N. Zabarska, D. Sorsche, F. W. Heinemann, S. Glump and S. Rau, *Eur. J. Inorg. Chem.*, **2015**, 2015, 4869-4877.
5. M. Nagao, K. Mundsinger and C. Barner-Kowollik, *Angew. Chem. Int. Ed.*, **2025**, *64*, e202419205.
